# Dominant Plant species and functional groups driving desert productivity changes in China and Egypt

**DOI:** 10.1016/j.isci.2026.116510

**Published:** 2026-06-30

**Authors:** Waqar Islam, Bo Zhang, Bingkun Yu, Qiang Yu, Haiyan Ren, Yongxin Zang, Abdelraouf A. Moustafa, Fanjiang Zeng

**Affiliations:** 1 State Key Laboratory of Ecological Safety and Sustainable Development in Arid Lands, Xinjiang Institute of Ecology and Geography, Chinese Academy of Sciences, Urumqi 830011, China; 2 Xinjiang Key Laboratory of Desert Plant Roots Ecology and Vegetation Restoration, Xinjiang Institute of Ecology and Geography, Chinese Academy of Sciences, Urumqi 830011, China; 3 Cele National Station of Observation and Research for Desert-Grassland Ecosystems, Cele 848300, China; 4 University of Chinese Academy of Sciences, Beijing 100049, China; 5 School of Grassland Science, Beijing Forestry University, Beijing 10083, China; 6 College of Agro-Grassland Science, Nanjing Agricultural University, Nanjing 210095, China; 7 Botany Department, Faculty of Science, Suez Canal University, Ismailia 41522, Egypt

**Keywords:** ecosystem productivity, plant functional groups, climate change, drought tolerance, functional traits, restoration ecology

## Abstract

Arid and semi-arid ecosystems in China and Egypt are increasingly threatened by climate change and human activities, making plant functional diversity critical for maintaining productivity and resilience. This review examines how dominant plant species and functional groups contribute to ecosystem functioning through traits such as photosynthetic pathways, water-use efficiency, nutrient acquisition, deep rooting, nitrogen fixation, and drought tolerance. Species including *Haloxylon ammodendron*, *Acacia tortilis*, and *Zygophyllum* spp. play key roles in sustaining ecosystem services under heat, salinity, and water scarcity. We highlight the contributions of C3, C4, and CAM plants to carbon cycling and ecosystem stability, and discuss climate-driven shifts toward drought-tolerant vegetation. The review also assesses the impacts of overgrazing, land-use change, and unsustainable management, and proposes trait-based restoration and climate-smart conservation strategies to enhance resilience and sustainable land management in arid regions.

## Graphical abstract

**Figure:**
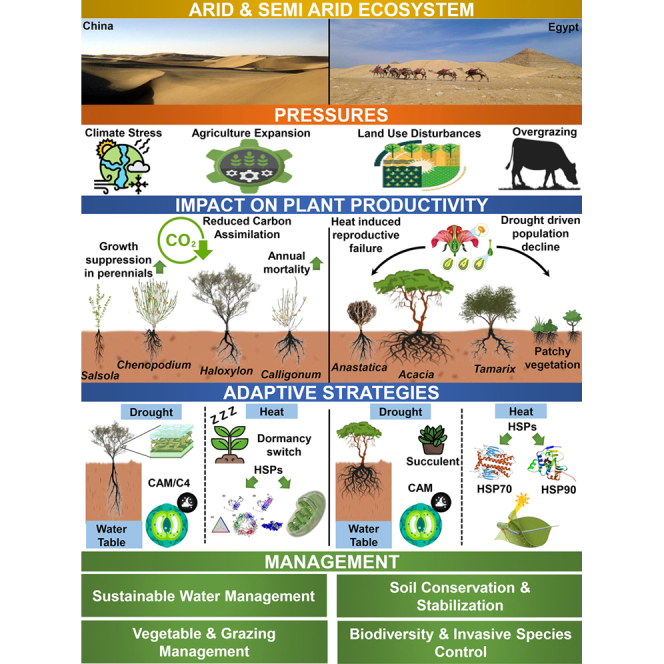

## Teaser

Environmental science; Ecology

## Introduction

Ecosystem productivity, the rate at which plants convert solar energy into biomass, is a fundamental driver of ecological stability and resilience. It regulates nutrient cycling, carbon sequestration, and overall ecosystem functioning.^1^^,^^2^ The contribution of individual plant species and functional groups to ecosystem productivity varies across environmental gradients, especially in arid and semi-arid regions where water availability is a major limiting factor.^3^ Understanding these contributions is critical in predicting ecosystem responses to environmental changes, particularly in the context of climate change.

China and Egypt, despite being geographically distant, share similarities in their desert and semi-arid ecosystems, both of which are highly vulnerable to climate extremes. In China, vast regions such as the Gobi and Taklamakan deserts experience significant temperature fluctuations, strong winds, and seasonal droughts, influencing vegetation dynamics.^4^^,^^5^ Similarly, Egypt’s arid landscapes, including the Sahara Desert and the Nile Basin, face extreme heat and prolonged droughts, further exacerbated by human activities such as land degradation and water resource exploitation.^6^^,^^7^ The productivity of these ecosystems depends largely on the ability of plant species and functional groups to adapt to harsh conditions.^8^ However, the relative contributions of different species and functional groups to ecosystem productivity in these regions remain underexplored.

Plant species play a crucial role in determining ecosystem productivity through their morphological, physiological, and biochemical adaptations.^9^ Certain species, such as deep-rooted perennials and nitrogen-fixing legumes, significantly influence soil fertility and water retention, while others, such as drought-resistant shrubs, contribute to carbon storage and erosion control.^10^ The classification of plants into functional groups—based on shared traits such as growth form, water-use efficiency, and photosynthetic pathways—provides a broader understanding of how vegetation structures influence productivity.^11^ For instance, C4 plants, which are more efficient in photosynthesis under high temperatures, often dominate arid environments,^12^ whereas C3 plants may be more sensitive to climatic fluctuations.^13^

Climate change is intensifying environmental stressors, leading to shifts in species composition and functional group dynamics.^14^ Rising temperatures, altered precipitation patterns, and increasing occurrences of extreme drought events threaten ecosystem productivity by disrupting plant physiological processes.^15^ In China, studies have shown that desert vegetation is undergoing compositional changes, with some species becoming more dominant while others decline.^16^ Similarly, in Egypt, declining water availability and soil degradation are affecting plant community structures, reducing overall biomass production.^17^ These changes have profound implications for biodiversity conservation, carbon cycling, and ecosystem services.^16^^,^^17^

**Figure 1:**
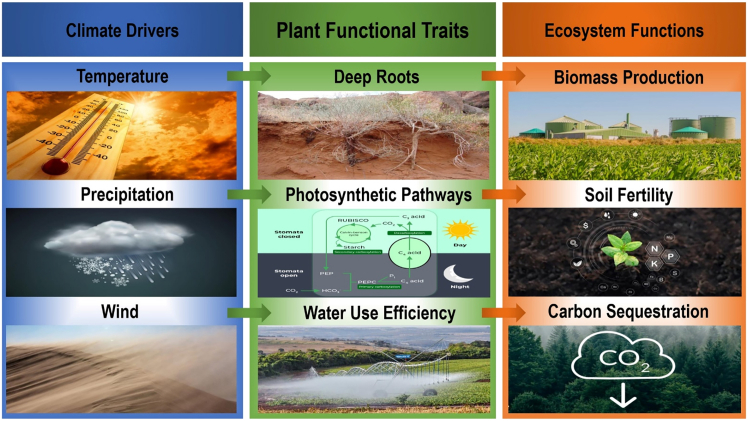
Conceptual framework diagram shows the relationship among climate drivers (temperature, precipitation, wind), plant functional traits (deep roots, photosynthetic pathways, water use efficiency), and ecosystem response (biomass production, soil fertility, carbon sequestration)

In addition to climate change, human activities such as overgrazing, deforestation, and unsustainable agricultural practices further impact ecosystem productivity.^18^ In both China and Egypt, the expansion of agricultural land into fragile ecosystems disrupts native plant communities and alters functional group distributions.^17^^,^^19^ Understanding the interactions between climate change, species composition, and functional groups is essential for developing sustainable land management strategies that enhance resilience and mitigate degradation (Figure 1).

This review aims to examine the relative contributions of plant species and functional groups to ecosystem productivity in China and Egypt. It will explore how different dominant plant species influence primary productivity, analyze the role of functional groups in ecosystem functioning, and assess the impacts of extreme climate events on vegetation dynamics. Additionally, it will discuss human-induced changes and propose management strategies for sustaining ecosystem productivity in these arid and semi-arid regions. By synthesizing current knowledge, this review will provide insights into the mechanisms driving vegetation responses to climate change and highlight key areas for future research.

## Methodology

### Review framework and design

This review was conducted following the preferred reporting items for systematic reviews and meta-analyses (PRISMAs 2020) guidelines to ensure transparency and reproducibility in literature selection and synthesis. The goal was to systematically identify, evaluate, and synthesize research examining the relative contributions of dominant and subdominant plant species and functional groups to desert ecosystem productivity in China and Egypt under changing climatic conditions.

### Literature search strategy

A comprehensive and systematic literature search was performed between January and June 2025 across major scientific databases: Web of Science (Core Collection), Scopus, ScienceDirect, SpringerLink, and Google Scholar. The search combined Boolean operators and keywords related to ecosystem productivity, functional groups, and desert vegetation in the target regions. The final search string used across databases was: (“ecosystem productivity” OR “primary productivity” OR “net primary productivity” OR “NPP” OR “GPP”) AND (“functional group” OR “plant functional trait” OR “species contribution” OR “dominant species” OR “subdominant species”) AND (“desert ecosystem” OR “arid ecosystem” OR “semi-arid ecosystem”) AND (“China” OR “Egypt” OR “Taklamakan” OR “Gobi” OR “Sahara” OR “Sinai” OR “Eastern Desert”) AND (“climate change” OR “drought” OR “heat stress” OR “aridity” OR “salinity” OR “desertification”). Reference lists of relevant reviews and empirical papers were manually screened to identify additional studies.

### Inclusion and exclusion criteria

Studies were selected based on clearly defined inclusion and exclusion criteria to minimize bias and ensure the relevance and reliability of the evidence base. Eligible studies published in English, including peer-reviewed empirical and review articles published between January 2000 and June 2025, focusing on arid and semi-arid ecosystems located in China or Egypt. Only research explicitly addressing plant species, functional groups, or functional traits in relation to ecosystem productivity, carbon or nutrient cycling, or resilience to climate stressors was considered. Studies utilizing field data, remote sensing, or experimental approaches were included to ensure methodological diversity and comprehensiveness.

Conversely, studies focusing exclusively on non-desert ecosystems—such as forests, wetlands, or humid grasslands—were excluded due to their limited relevance to arid environments. Publications lacking empirical or quantitative data, such as editorials, short communications, or commentaries, were also excluded. Additionally, studies that did not clearly link vegetation traits to ecosystem productivity were omitted. Duplicate entries and inaccessible full-text documents were removed during the screening process. Publications available solely in Chinese or Arabic without English translation were excluded to ensure accuracy and comparability in data interpretation.

### Study selection and screening process

All retrieved records were imported into mendeley reference manager for systematic organization and duplicate removal. The screening process followed two key stages to ensure accuracy and transparency. In the first stage, titles and abstracts were screened to exclude studies that were clearly irrelevant to the research scope. In the second stage, full-text articles were reviewed to confirm their relevance, methodological soundness, and completeness of data. The screening process was independently conducted by two reviewers, with disagreements resolved through discussion or, when necessary, by a third reviewer to maintain objectivity. The overall selection process resulted in 988 identified records, out of which 871 were screened, 637 excluded, and ultimately 234 were included in the final synthesis. This systematic approach ensured that only high-quality, relevant studies were included in the review.

### Data extraction and synthesis

For each included study, critical data were systematically extracted using a structured template designed to capture the most relevant ecological and methodological details. The extracted information included the study location and ecosystem type, the dominant and subdominant plant species investigated, and the functional group classification (e.g., C3, C4, CAM, legume, shrub, or grass). Additionally, key indicators of ecosystem productivity—such as net primary productivity (NPP), gross primary productivity (GPP), biomass accumulation, or carbon flux—were recorded. Contextual environmental drivers, including precipitation, temperature, salinity, and land use practices, were also documented, along with any reported adaptive traits or ecological functions relevant to productivity or stress tolerance.

The extracted data were qualitatively synthesized to identify overarching patterns in species contributions and trait-productivity relationships between China and Egypt. Where available, quantitative data such as productivity metrics or climate-productivity correlations were summarized descriptively. However, due to methodological variability and data heterogeneity among studies, a formal meta-analysis was not conducted. This synthesis allowed for a comparative understanding of how different plant functional traits and groups shape desert ecosystem productivity under varying environmental pressures.

### Quality assessment of included studies

To ensure the robustness and reliability of findings, the methodological quality of each included study was evaluated using a modified critical appraisal skills program (CASP) checklist, adapted for ecological and environmental research. The appraisal considered six key aspects: (1) clarity of research objectives and hypotheses, (2) adequacy of sampling design and replication; (3) transparency in data collection and analytical methods, (4) appropriateness of measurements related to productivity and functional traits, (5) relevance to desert ecosystems in China or Egypt, and (6) peer-review status and citation frequency as indicators of scientific credibility.

Each study was scored on a five-point scale (1 = poor, 5 = excellent). Only studies achieving a score of three or higher were included in the final synthesis. This assessment framework provided a transparent and systematic approach to evaluating study quality, ensuring that conclusions drawn from the review were supported by reliable evidence. The quality ratings were used to weigh interpretations in the discussion, giving greater confidence to findings derived from higher-quality studies.

### Limitations of the review process

While the present review employed a comprehensive and systematic approach, several limitations should be acknowledged. First, the exclusion of non-English and non-indexed local studies may have introduced a language bias, potentially overlooking regionally relevant research published in Chinese or Arabic. Second, differences in methodological approaches—including variations in productivity measurement techniques and sampling scales—limit direct quantitative comparisons across studies. Third, publication bias toward positive or significant results may have influenced the overall perception of plant trait-productivity relationships in desert ecosystems.

Despite these limitations, the transparent and reproducible methodology employed—including explicit inclusion and exclusion criteria, independent screening, and structured quality assessment—ensures a balanced and credible synthesis. This review provides a rigorous foundation for understanding plant functional contributions to desert ecosystem productivity and establishes a framework for future empirical and meta-analytical research.

## Ecosystem productivity, functional groups and plant species contributions to primary productivity and nutrient cycling

Ecosystem productivity in arid landscapes reflects the combined influence of plant functional traits, species composition, and resource limitation. In the deserts of China and Egypt, primary productivity and nutrient cycling are strongly shaped by the relative roles of dominant species and functional groups adapted to water and nutrient scarcity. Assessing how productivity is measured, how functional groups structure ecosystem processes, and how individual species regulate carbon and nutrient fluxes is critical for understanding vegetation responses to climate stress. This section integrates these dimensions to clarify productivity controls in desert ecosystems.

### Ecosystem productivity and its measurement

Ecosystem productivity refers to the rate at which plants and other photosynthetic organisms capture solar energy and convert it into organic biomass. It is typically categorized into primary productivity, which includes GPP and NPP.^20^ GPP represents the total amount of carbon fixed by plants through photosynthesis,^21^ while NPP is the portion of GPP that remains after plants use some of this energy for their metabolic processes (respiration), effectively contributing to biomass production.^22^ NPP is a critical measure of ecosystem function, as it represents the energy available for higher trophic levels, such as herbivores and decomposers. It is often measured through remote sensing techniques, biomass sampling, and carbon flux measurements *in situ*.^23^

### Functional groups: classification and ecological significance

Functional groups are collections of plant species that share similar ecological roles or functional traits, such as photosynthetic pathway, water-use efficiency, or growth form. These traits allow functional groups to perform comparable ecosystem functions, even if the species within them are taxonomically unrelated.^24^^,^^25^ For example, C3 plants, which use the Calvin cycle for photosynthesis, and C4 plants, which utilize a more efficient pathway in high-temperature environments, are both critical in shaping ecosystem productivity and carbon cycling in arid ecosystems.^26^ The classification of plants into functional groups helps predict ecosystem responses to environmental changes, as species with similar traits will likely respond in comparable ways to climatic stressors.^27^^,^^28^ Functional groups contribute to ecosystem services such as nutrient cycling, soil stabilization, and biodiversity maintenance.^29^ For instance, leguminous plants, a functional group known for their nitrogen-fixing ability, play an essential role in enhancing soil fertility, while deep-rooted perennial grasses can improve water retention and reduce soil erosion.^30^

### Plant species contributions to primary productivity and nutrient cycling

Each plant species contributes differently to primary productivity and nutrient cycling, depending on its specific traits. For example, deep-rooted plants can access water and nutrients from deeper soil layers, contributing to the overall productivity of an ecosystem, particularly during periods of drought.^31^ Additionally, plants that are highly efficient in water use, such as xerophytes (plants adapted to dry conditions), can survive and maintain productivity in arid environments. These species are vital for sustaining ecosystem processes in the face of limited water availability.^32^

Nutrient cycling is also significantly influenced by plant species. For example, nitrogen-fixing plants (e.g., legumes) can enhance soil fertility by converting atmospheric nitrogen into a form that other plants can utilize.^33^ This ability is crucial in ecosystems where nitrogen availability is a limiting factor for plant growth. Furthermore, plant species contribute to carbon cycling through the capture and storage of carbon in both above-ground biomass and soil organic matter, thus influencing the global carbon budget.^33^^,^^34^

## Climatic and environmental conditions in China and Egypt

Climatic and environmental conditions are the primary constraints shaping ecosystem productivity in arid and semi-arid regions. In China and Egypt, extreme temperature regimes, low and variable precipitation, intense solar radiation, and strong winds interact to regulate vegetation structure, species distribution, and primary productivity. This section examines key climatic drivers, compares environmental contexts between the two regions, and evaluates how ongoing climate change is reshaping plant communities and ecosystem functioning.

### Key climatic factors affecting ecosystem productivity

Climatic factors—particularly temperature, precipitation, solar radiation, and wind—play a critical role in regulating ecosystem productivity in desert and semi-arid environments.^35^^,^^36^ In both China and Egypt, where deserts such as the Taklamakan, Gobi, Sahara, and Sinai Peninsula dominate the landscape, the productivity of ecosystems is tightly constrained by water availability and thermal extremes.^37^^,^^38^

Temperature is one of the most important factors shaping ecosystem productivity. It affects the rate of photosynthesis, respiration, and overall metabolic processes of plants.^39^ In China’s Taklamakan Desert, average annual temperatures hover around 12 °C, but seasonal extremes range from below −26 °C in winter to over 40 °C in summer, placing considerable stress on both plants and soil microbes.^40^ The Gobi Desert presents even harsher fluctuations, with winter temperatures dropping to −40 °C and summer peaks nearing 45 °C, accompanied by daily thermal swings of up to 35 °C.^4^ These extremes challenge plant survival, reduce microbial activity, and limit the growing season. In Egypt, the Sinai Peninsula’s higher elevations remain relatively cool, while lowland areas often exceed 30 °C during summer.^41^ The Sahara Desert, with temperatures surpassing 47 °C and rainfall rarely exceeding 50–100 mm/year, represents one of the most thermally and hydrologically constrained ecosystems on Earth.^42^

Precipitation plays a pivotal role in determining ecosystem productivity, especially in water-limited environments.^43^ In both the Taklamakan and Gobi, annual rainfall ranges between 30 and 70 mm, with hyper-arid interiors receiving less than 10 mm. Nevertheless, recent years have shown episodic summer precipitation surges—e.g., +36 mm in a single month in oasis zones—suggesting the increasing impact of extreme events.^4^^,^^44^ In the Sinai Peninsula, rainfall is topographically influenced: mountain wadis receive intermittent rain, while most areas receive <25 mm/year, leading to persistent drought conditions.^45^ The Sahara Desert remains the driest among them, with some regions experiencing years without any rainfall, severely restricting primary productivity.^46^

Solar radiation is a primary driver of photosynthesis, providing the energy necessary for plant growth.^47^ In desert ecosystems, the intensity of solar radiation is often high, but productivity is constrained by water and temperature limitations. The efficiency with which plants capture and utilize solar energy can differ significantly between species, with some species having adapted mechanisms to optimize photosynthesis under intense sunlight.^48^

Wind is another climatic factor influencing ecosystem productivity, particularly in desert and semi-arid regions.^49^ In the Gobi, strong winds influenced by the Siberian anticyclone cause frequent sandstorms, contributing to erosion and soil degradation.^50^ In the Taklamakan Desert, sandstorms exacerbate land degradation by stripping away nutrient-rich topsoil.^51^ Similarly, Egypt’s Sinai and Sahara deserts are impacted by seasonal wind events such as the “khamsin,” which erode exposed surfaces, reduce soil moisture, and damage already sparse vegetation.^52^

Together, these climatic factors create a dynamic environment that shapes the productivity of desert and semi-arid ecosystems. Understanding the interactions between temperature, precipitation, and other climatic drivers is essential for evaluating the resilience of plant communities to future climate shifts.

### Comparison of arid and semi-arid ecosystems in China and Egypt

**Figure 2:**
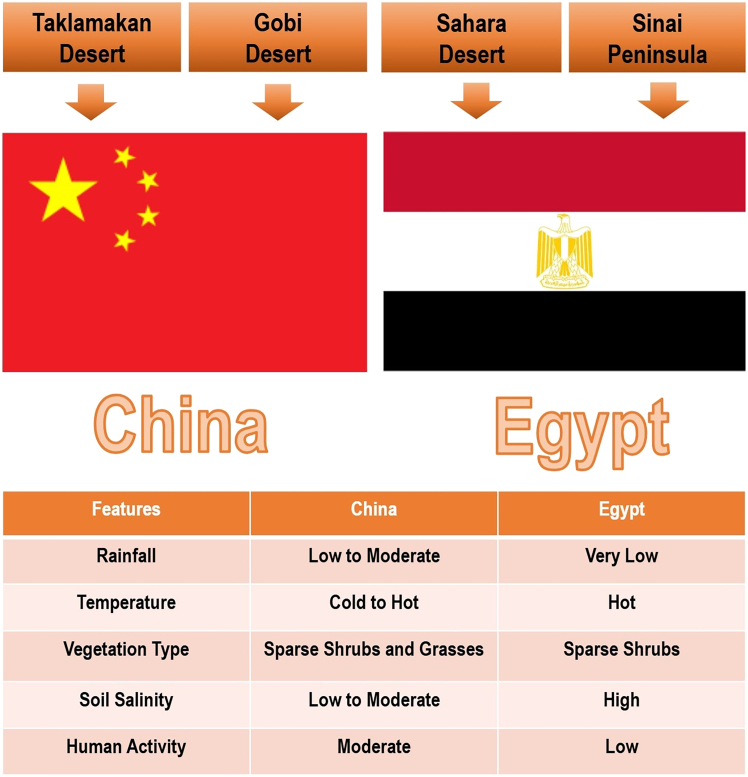
Side-by-side environmental comparison of China and Egypt

China and Egypt, though located in different parts of the world, share significant similarities in their arid and semi-arid ecosystems. Both countries contain vast desert areas with limited rainfall and extreme climatic conditions, making them highly vulnerable to changes in climate (Figure 2; Table 1).

**Table 1:** Key climatic and environmental conditions in China and Egypt

Aspect	China	Egypt	Similarities	Differences	Implications for ecosystem productivity	Reference
Major deserts	Gobi Desert, Taklamakan Desert, Qinghai-Tibet Plateau	Sahara Desert, Eastern Desert	both have vast arid and semi-arid regions	China has colder winters; Egypt has more extreme heat	productivity is limited by extreme temperatures and water scarcity in both regions	El-Agha et al.,^6^ El-Ramady et al.^53^
Climate	extreme temperature fluctuations, seasonal droughts, low rainfall	extreme heat, prolonged droughts, sporadic rainfall	both face water scarcity and temperature extremes	China has more seasonal variability; Egypt has more consistent high temperatures	seasonal droughts in China reduce productivity; Egypt’s consistent heat stresses vegetation year-round	Elkhouly et al.,^54^ Chen et al.^55^
Precipitation	highly variable, mostly in summer (e.g., <50 mm annually in Taklamakan)	minimal, sporadic (e.g., Sahara Desert)	both have low and unpredictable rainfall	China’s rainfall is more seasonal; Egypt’s is more sporadic	low rainfall limits plant growth, but seasonal rains in China allow for brief productivity bursts	Nashwan et al.,^46^ Li et al.^56^
Temperature	cold winters, hot summers (e.g., >40°C in summer, below freezing in winter)	extreme heat (e.g., >40°C in summer)	both experience extreme temperatures	China has colder winters; Egypt has more consistent high temperatures	cold winters in China limit plant growth; Egypt’s heat reduces photosynthesis and increases water loss	Chen et al.,^55^ Eid et al.^57^
Vegetation	sparse, drought-tolerant grasses, shrubs, and hardy trees (e.g., poplar)	sparse, drought-resistant shrubs and trees (e.g., Acacia, Zygophyllum)	both have vegetation adapted to extreme drought conditions	China has more cold-tolerant species; Egypt has more heat-tolerant species	vegetation in both regions is adapted to survive, but productivity is low due to harsh conditions	El-Amier,^58^ Zhu et al.^59^
Human Pressures	overgrazing, deforestation, agricultural expansion, desertification	overgrazing, agricultural expansion, water resource exploitation	both face significant human-induced land degradation	China’s desertification is driven by colder climates; Egypt’s by extreme heat	human activities exacerbate natural stressors, further reducing ecosystem productivity	Bedair et al.,^17^ Liu & Xin^60^
Key Adaptations	deep-rooted plants, drought-resistant shrubs, nitrogen-fixing species	deep-rooted trees, succulent plants, salt-tolerant species	both have species adapted to water scarcity and extreme temperatures	China’s species are more cold-adapted; Egypt’s are more heat-adapted	adaptations help species survive, but productivity remains low due to extreme environmental conditions	Salama et al.,^61^ Qian et al.^62^

In China, the most notable arid and semi-arid regions include the Gobi Desert, Taklamakan Desert, and parts of the Qinghai-Tibet Plateau.^63^ These regions are characterized by low annual rainfall, high evaporation rates, and significant temperature fluctuations.^64^ The Gobi Desert, for example, experiences extremely cold winters and hot summers, which influence plant growth and ecosystem dynamics. Precipitation is highly variable, with most of it occurring in the summer months, resulting in seasonal droughts. Vegetation in these areas is typically sparse, consisting mainly of drought-tolerant grasses, shrubs, and some hardy tree species, such as poplar and willow. The plant species in these ecosystems have evolved specific adaptations to survive harsh conditions, such as deep root systems to access groundwater and mechanisms for conserving moisture.^4^^,^^65^^,^^66^

In Egypt, the most prominent desert regions include the Sahara Desert and the Eastern Desert, which are similarly marked by low rainfall and extreme temperature variations. The Nile River plays a crucial role in maintaining productivity along its banks, with irrigation systems supporting agriculture in an otherwise arid landscape.^54^ Outside the Nile Valley, the Sahara is extremely dry, with the majority of rainfall occurring in sporadic events.^67^ The Eastern Desert, which is more mountainous, experiences cooler temperatures and occasional rainfall, but it is still considered an arid environment.^68^ Vegetation in Egypt’s desert regions is sparse, with species such as acacia, date palms, and various shrubs adapted to the harsh conditions.^54^ Like their counterparts in China, plants in these regions are adapted to conserve water and withstand extreme temperatures.

Both countries also face pressures from human activities such as agriculture, urbanization, and land degradation. In China, desertification has been exacerbated by agricultural expansion, deforestation, and overgrazing, particularly in regions such as Inner Mongolia.^69^ In Egypt, the expansion of agricultural lands along the Nile and the use of water for irrigation have altered the natural balance of ecosystems.^70^ Both regions are vulnerable to desertification, which is further compounded by climate change, increasing the need for effective land management and conservation strategies.

### Impacts of climate change on plant communities and productivity in China and Egypt

Climate change is expected to have profound effects on ecosystem productivity, particularly in arid and semi-arid regions such as China and Egypt. Rising temperatures, altered precipitation patterns, and increased frequency of extreme climate events (e.g., droughts and heatwaves) are likely to exacerbate existing stresses on plant communities and influence overall productivity.^71^

In China, studies have shown that increasing temperatures and changes in precipitation patterns have led to shifts in vegetation composition, with some species becoming more dominant while others decline. For instance, species that are adapted to cold conditions may be displaced by warmer-adapted species as temperatures rise. Lu et al.^72^ reported that the invasive plant *Alternanthera philoxeroides* has been expanding its range northwards in China due to warmer winters, suggesting that it can tolerate cold better than its natural enemies. This shift may lead to a geographical gap between invasive plants and their herbivorous insects, resulting in a new zone of enemy release and potentially altering community structures. Moreover, changes in precipitation are expected to shorten growing seasons and reduce plant diversity, limiting the establishment of stable vegetation cover. For instance, He et al.^73^ used an *in situ* experiment in a desert-grassland region of northern China to simulate different precipitation amounts and intervals. They found that both precipitation quantity and timing significantly influenced *Artemisia ordosica* community structure. Increased precipitation boosted species diversity by enhancing perennial forb richness, while longer intervals increased diversity under low precipitation but reduced it under high precipitation. Overall, precipitation patterns shaped community composition and diversity primarily through their impact on soil moisture. On a similar note, drought-induced reductions in water availability may decrease the abundance of moisture-dependent species such as grasses and forbs, while favoring the dominance of drought-tolerant shrubs and deep-rooted plants. Yao et al.^74^ conducted a large-scale quantitative analysis across 1,039 quadrats at 184 sites in China’s drylands to assess the relative influence of water and energy on plant diversity. Their findings highlighted that water availability, along with its interaction with energy, plays a critical role in shaping plant diversity—supporting the predictions of the water-energy dynamics hypothesis.

In Egypt, rising temperatures are expected to accelerate evaporation rates, reduce surface water availability, and place greater stress on plant communities. Desert regions outside the Nile Valley are especially vulnerable to increased aridification, which may drive the expansion of desert areas and threaten existing ecosystems.^75^ Species dependent on shallow groundwater or surface moisture are likely to be most affected, resulting in shifts in species composition and declining ecosystem productivity. Yassen et al.^76^ investigated the spatiotemporal patterns of annual and monthly reference evapotranspiration across Egypt over a 35-year period. Their findings revealed significant changes in evapotranspiration distribution since the 1980s, with the southeastern regions, older agricultural lands in the Nile Delta and Valley, and the northwestern areas experiencing the most pronounced impacts. On the other hand, some drought-tolerant species may thrive under these new conditions, although they may not fully compensate for the loss of more productive plant species.^32^

Both China and Egypt are also likely to face more frequent extreme climate events, such as prolonged droughts or heatwaves, which can have immediate and severe impacts on plant productivity. These events can lead to a reduction in biomass production, dieback of vulnerable species, and shifts in community structure. In desert regions, where ecosystems are already at the edge of survival, such events could have cascading effects on ecosystem services such as carbon sequestration, soil fertility, and water retention.

## Relative contributions of plant species to ecosystem productivity in China and Egypt

Ecosystem productivity in arid and semi-arid regions is not evenly distributed among plant species but is disproportionately driven by a limited number of dominant taxa and functional groups. In the deserts of China and Egypt, species-specific traits, stress-tolerance strategies, and interspecific interactions determine how plants contribute to carbon fixation, nutrient cycling, and ecosystem stability. This section synthesizes evidence on the relative contributions of key plant species, their responses to environmental stressors, and the functional traits and group interactions that regulate productivity under extreme climatic conditions. Importantly, comparing China and Egypt reveals that similar ecosystem functions—such as productivity maintenance under drought—are often achieved through phylogenetically distinct lineages, suggesting convergent functional outcomes but potentially divergent ecosystem processes and resilience pathways.

### Dominant plant species and their ecological roles

In arid and semi-arid ecosystems, dominant plant species play a critical role in shaping ecosystem structure, functioning, and productivity. These species not only contribute to primary productivity but also influence soil structure, nutrient cycling, water retention, and biodiversity.^77^ Dominant plants in desert ecosystems tend to be highly specialized to withstand extreme environmental conditions, including prolonged droughts, temperature fluctuations, and poor soils. Their survival and persistence often depend on a suite of morphological, physiological, and biochemical adaptations that allow them to minimize water loss, maximize nutrient uptake, and optimize energy storage during short growing seasons.^78^^,^^79^

**Figure 3:**
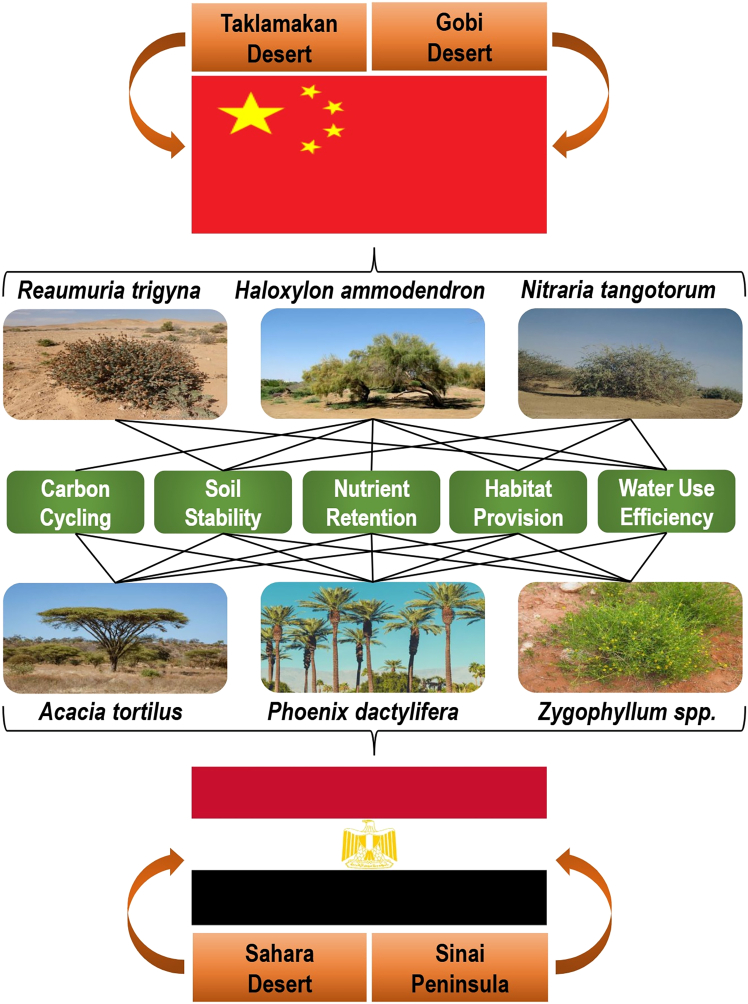
Dominant plant species in China and Egypt and their ecological roles In the Taklamakan and Gobi deserts, dominant species such as *Reaumuria trigyna*, *Haloxylon ammodendron*, and *Nitraria tangutorum* play a vital role in carbon cycling, soil stability, nutrient retention, habitat provision, and water use efficiency. Similarly, in the Sinai Peninsula and the Sahara Desert of Egypt, key plants such as *Acacia tortilis*, *Phoenix dactylifera*, and *Zygophyllum* species perform these same crucial ecological functions.

In China, desert ecosystems such as the Taklamakan and Gobi Deserts are characterized by the dominance of drought-tolerant shrubs, hardy grasses, and a few resilient tree species. In the Taklamakan Desert, key plant species include *Alhagi sparsifolia*, a deep-rooted leguminous shrub common in interdune depressions and river margins; *Tamarix ramosissima*, a salt-tolerant shrub or small tree typically found along riverbanks and saline soils; *Karelinia caspia*, a perennial herb/shrub thriving in sandy and saline habitats; *Calligonum mongolicum*, adapted to mobile dunes and arid plains; *H. ammodendron* (Saxaul tree), a dominant drought-tolerant species in dune and gravel regions; *Reaumuria soongarica*, a woody shrub frequent on saline and gravelly soils; *Nitraria tangutorum*, a halophytic shrub found in salt-affected and semi-fixed dunes; and *Populus euphratica*, typically occurring in riverine oases associated with groundwater.^80^ In the Gobi Desert, dominant species include *N. tangutorum* and *R. soongarica*, both widely distributed and highly drought-resistant; *H. ammodendron*, prevalent in arid and sandy areas; *Anabasis brevifolia*, a dwarf shrub covering gravel plains; Artemisia species (e.g., *A. frigida*, *A. desertorum*), which are typical of semi-desert and steppe environments; *Salsola passerina*, a shrub well adapted to saline and alkaline soils; *Z. xanthoxylum*, a perennial suited to arid, gravel-rich terrain; and *Ephedra przewalskii*, a woody shrub capable of surviving in hyper-arid conditions^81^ (Figure 3; Table 2).

**Table 2:** Dominant plant species and their ecological roles in China and Egypt

Country	Dominant species	Ecological role	Adaptations	Contribution to ecosystem productivity	Response to climate stress	Reference
China	*Haloxylon ammodendron*	deep-rooted shrub, stabilizes soil, reduces erosion, enhances carbon cycling	deep roots, waxy leaves	high carbon sequestration, soil stabilization, supports other species	resilient to drought and temperature fluctuations; growth slows under extreme stress	Von Wehrden et al.,^81^ Li et al.^82^
	*Reaumuria soongarica*	drought-resistant shrub, soil stabilization, protects surface crust	small, scale-like leaves, salt tolerance	prevents wind erosion, supports microhabitats, enhances soil structure	tolerant of drought and salinity; sensitive to repeated frost or heat extremes	
	*Nitraria tangutorum*	nitrogen-fixing shrub, improves soil fertility, supports other species	deep roots, salt and drought tolerance	enhances soil fertility, promotes biodiversity, aids in dune stabilization	highly drought- and salt-tolerant; growth declines in extreme cold	
	*Alhagi sparsifolia*	nitrogen-fixing legume, stabilizes dunes, improves fertility	deep taproot, salt/drought tolerance	improves nutrient cycling, supports microbial activity, facilitates other species	highly resilient to drought; leaf shedding under severe heat or cold stress	
	*Tamarix ramosissima*	riparian buffer species, controls salinity, provides habitat	salt glands, deep roots, drought tolerance	stabilizes riverbanks, reduces salinization, supports bird and insect populations	tolerant of salinity and drought; vulnerable to groundwater drop	
	*Karelinia caspia*	herb/shrub stabilizing saline soils	halophytic traits, perennial	binds soil, improves microhabitats in saline areas	tolerant of salt and moderate drought; sensitive to disturbance	
	*Calligonum mongolicum*	sand dune stabilizer, supports early colonization	flexible stems, xerophytic leaves	critical for early succession, reduces sand movement	resilient to sand burial and drought; suffers under prolonged heat	
	*Populus euphratica*	canopy cover in oases, prevents desert encroachment	phreatophytic roots, salt tolerance	Enhances biodiversity, hydrological cycles, habitat provider	Sensitive to groundwater depletion; resilient to moderate drought	
	*Anabasis brevifolia*	covers gravel plains, suppresses evaporation	compact size, thickened tissues	Stabilizes surface soil, supports forage availability	Tolerates aridity; growth is slow under extremes	
	*Artemisia* spp.	ground cover, medicinal and forage species	hairy leaves, volatile oils	Supports biodiversity, erosion control, suppresses weeds	Drought-tolerant; some species sensitive to heatwaves	
	*Salsola passerina*	salt-tolerant shrub, improves degraded soils	succulent leaves, extensive roots	Enhances marginal land productivity, stabilizes saline soils	Highly tolerant of salinity; moderately heat-sensitive	
	*Zygophyllum xanthoxylum*	gravel-soil shrub, aids water retention	succulent leaves, CAM metabolism	Maintains productivity in harsh soils, supports microbial life	Thrives in drought; growth reduces under salinity/heat extremes	
	*Ephedra przewalskii*	woody shrub, prevents erosion, has medicinal value	reduced leaves, green stems	Structural diversity, traditional medicine, soil stabilizer	Tolerant to aridity; vulnerable to long-term moisture loss	
Egypt	*Acacia tortilis*	deep-rooted tree, stabilizes soil, provides shade	deep roots, heat and drought tolerance	Supports biodiversity, improves soil structure, enhances carbon storage	Very drought-tolerant; growth declines with prolonged water scarcity	Amro et al.,^83^ Abbas et al.^84^
	*Tamarix nilotica*	salt-tolerant shrub, stabilizes saline soils, riparian species	salt glands, deep roots	Manages salinity, habitat for birds and insects	Tolerant of salinity and drought; sensitive to groundwater depletion	
	*Zygophyllum coccineum*	drought-resistant shrub, stabilizes soil, supports fauna	succulent leaves, water storage	Ground cover, nutrient cycling, forage source	Thrives in arid and saline conditions; heat-sensitive	
	*Panicum turgidum*	perennial grass, dune stabilizer	extensive fibrous roots	Reduces erosion, supports microhabitats	Tolerant of drought and sand movement; slow recovery in prolonged drought	
	*Alhagi maurorum*	legume shrub, improves soil fertility	nitrogen-fixing, salt tolerance	promotes soil productivity and microbial activity	resilient to drought and salinity	
	*Calligonum comosum*	early colonizer of desert sands	flexible stems, wind resistance	stabilizes sand, supports biodiversity	tolerates burial and drought; reduced growth under heat stress	
	*Haloxylon salicornicum*	shrub of gravel plains, reduces erosion	leaf reduction, thick stems	soil stabilizer, supports crust formation	tolerant to drought/salinity; sensitive under extreme aridity	
	*Balanites aegyptiaca*	tree in wadis, provides food and shade	deep taproot, drought tolerance	supports wildlife and people, enhances habitat diversity	drought-resilient; sensitive to overharvest and extreme dry periods	
	*Zilla spinosa*	thorny shrub, erosion control	spiny structure, drought resistance	forage in arid zones, binds surface soil	hardy under drought; sensitive to temperature extremes	
	*Fagonia* spp.	medicinal herb/shrub in arid zones	prostrate growth, low water needs	enhances diversity, traditional use	arid-adapted; sensitive to increasing heat extremes	
	*Maerua crassifolia*	shrub/tree in deep wadis, provides forage	deep roots, heat resistance	pastoral resource, supports soil moisture	tolerates heat; vulnerable to overuse	
	*Leptadenia pyrotechnica*	shrub stabilizing sandy deserts	leafless, stem photosynthesis	critical for erosion control, aids seedling establishment	highly drought- and heat-resilient	
	*Anabasis articulata*	shrub in gravel plains, improves soil retention	salt/drought-tolerant, compact form	reclaims degraded lands, supports soil structure	withstands poor soils and aridity; sensitive to grazing	
	*A. judaica*	medicinal/forage shrub, supports pollinators	aromatic leaves, low water need	medicinal resource, promotes insect biodiversity	drought-tolerant; temperature-sensitive	
	*Thymelaea hirsuta*	soil stabilizer and medicinal shrub	hairy leaves, deep-rooted	rock soil binder, supports ecological balance	drought-resilient; slow growth under heat extremes	
	*Retama raetam*	nitrogen-fixing shrub, provides habitat	deep roots, drought tolerance	improves fertility, offers microhabitat	highly drought-tolerant; sensitive to fragmentation	
	*Achillea fragrantissima*	aromatic shrub, medicinal and pollinator-friendly	water-efficient metabolism	supports livelihoods, pollination services	arid-adapted; overharvest-sensitive	
	*Gymnocarpos decandrum*	soil stabilizer in rocky deserts	minimal water needs, compact form	reduces erosion, promotes cover	arid-tolerant; sensitive to prolonged drought	
	*Capparis spinosa*	spiny shrub, edible and medicinal, stabilizes rocks	succulent leaves, deep roots	enhances diversity, supports traditional uses	very drought-tolerant; frost-sensitive	

Although dominant shrubs in both regions perform broadly similar roles in carbon fixation, soil stabilization, and water regulation, they originate from contrasting evolutionary lineages. Chinese deserts are largely dominated by Chenopodiaceae (e.g., *Haloxylon*, *Salsola*, *Reaumuria*), which evolved in cold-desert systems with strong seasonal temperature constraints, whereas Egyptian deserts are dominated by Fabaceae (*Acacia*) and Zygophyllaceae (*Zygophyllum*), lineages shaped by hot-desert evolution. This phylogenetic divergence has important functional consequences for nutrient cycling, physiological plasticity, and long-term ecosystem resilience.

Similarly, In Egypt, the dominant plant species across arid regions such as the Sahara Desert (Egyptian part), Eastern Desert, and Sinai Peninsula exhibit a range of drought- and salt-tolerant adaptations that enable survival in extreme environments. In the Sahara Desert, common species include *A. tortilis*, a deep-rooted tree found in wadis; *T. nilotica*, a salt-tolerant shrub along saline soils; *Z. coccineum*, a widespread drought-resistant shrub; *Panicum turgidum*, a perennial grass that stabilizes sand dunes; *A. maurorum*, a leguminous shrub thriving in disturbed or saline zones; and *C. comosum* and *H. salicornicum*, both adapted to sandy or gravel plains.^85^ The Eastern Desert, characterized by rocky mountains and wadis, supports species such as *Balanites aegyptiaca*, *Zilla spinosa*, Fagonia spp., *Maerua crassifolia*, *Leptadenia pyrotechnica*, and *A. articulata*, all of which are highly adapted to arid, nutrient-poor soils.^86^ In the Sinai Peninsula, which harbors greater ecological diversity, dominant species include *A. judaica*, *Thymelaea hirsuta*, *Retama raetam*, *Achillea fragrantissima*, *Gymnocarpos decandrum*, and *Capparis spinosa*, with *Tamarix* spp. also common in saline depressions and wadis. These species play essential roles in soil stabilization, nutrient cycling, and supporting biodiversity under increasingly challenging climatic conditions^87^ (Figure 3; Table 2).

Plants in arid and semi-arid ecosystems, such as the Taklamakan and Gobi Deserts in China and the Sinai Peninsula in Egypt, have developed a wide range of physiological, morphological, and biochemical strategies to withstand harsh environmental conditions. These conditions include water scarcity, extreme temperatures, high salinity, and nutrient limitations. Such adaptations are essential for maintaining plant productivity and ensuring the long-term stability of desert ecosystems.^88^ One of the most important drought survival strategies is the development of deep root systems. These roots enable plants to access water stored deep underground, often far beyond the reach of shallow-rooted vegetation. In China’s deserts, *H. ammodendron* has been identified as a classic deep-rooted phreatophyte capable of drawing water from both shallow and deep groundwater reserves. Research has shown that this species can uptake water at night, contributing around 12% to its daily water use—an effective adaptation to limit water loss through evapotranspiration during hot days. In contrast, *C. mongolicum* primarily relies on mid- and shallow-layer soil moisture.^89^ Similarly, in Egypt’s Sinai Peninsula, *A. tortilis* and its subspecies have root systems that exceed 30 m in depth. These roots allow the trees to persist year-round in environments receiving less than 50 mm of annual rainfall by tapping into groundwater in wadis and mountainous areas.^90^ These contrasting rooting and water-use strategies suggest that Chinese desert systems may be more vulnerable to long-term groundwater decline, whereas Egyptian systems—dominated by fewer but functionally multifunctional trees such as *Acacia*—may exhibit greater short-term stability but higher sensitivity to loss of keystone species.

Water conservation through leaf morphology is another key adaptation. Many desert plants possess small, thick, waxy, or mucilage-rich leaves that minimize water loss through transpiration. In China’s Gobi Desert and Hexi Corridor, species in the Zygophyllaceae family—such as *Zygophyllum* spp.—are well known for their waxy cuticles and mucilage-secreting tissues, which help retain moisture and protect photosynthetic tissues during extreme drought.^91^ In Egypt, *A. tortilis* features small, bipinnate leaves that significantly reduce surface area for water loss. Physiological studies indicate that this species begins to close its stomata when soil volumetric water content falls below 9.8%, helping conserve internal water during extended dry periods.^92^ In addition to morphological traits, desert plants exhibit physiological mechanisms that enhance drought tolerance. A comparative study conducted in the Sinai Peninsula between *A. tortilis* and *A. raddiana* demonstrated that both species can maintain about 25% of their photosynthetic activity at soil volumetric water contents between 5 and 9%. Notably, *A. tortilis* uses a “water-spending” strategy by sustaining photosynthesis during midday heat and showing rapid recovery post-drought. This contrasts with *A. raddiana*, which employs a more conservative water-use approach, indicating differing survival strategies even among closely related species.^92^

Another critical trait observed in desert flora is the dual tolerance to drought and salinity, particularly relevant in saline groundwater zones. In the Taklamakan Desert, salt-tolerant species such as *C. mongolicum* and *H. ammodendron* have been successfully planted under saline drip irrigation conditions, with salinities exceeding 20 g/L. These species showed over 65% seedling survival and developed strong root systems, highlighting their suitability for reclamation and shelterbelt projects in hyper-arid, saline environments.^89^ In Egypt, *A. tortilis* not only tolerates arid and saline conditions but also plays a key role in improving soil structure. It enhances water infiltration and reduces soil erosion in mountainous wadis, contributing to landscape stabilization and supporting surrounding biodiversity, as demonstrated in ecological models of the Red Sea Hills.^93^ Thus, while both regions converge on drought-salinity tolerance as a prerequisite for dominance, Chinese shrub-dominated systems emphasize structural persistence and soil stabilization, whereas Egyptian tree-shrub systems integrate hydrological buffering with biogeochemical enhancement, particularly nitrogen inputs.

Overall, these adaptations reflect the ecological resilience of desert plants in both China and Egypt. Understanding their adaptive mechanisms is vital for informing sustainable land management, ecological restoration, and climate adaptation strategies in desert regions facing increased drought intensity and land degradation.

### Species-specific responses to environmental stressors

Photosynthetic pathways also play a significant role in species-specific responses to stress. Plants in arid regions often utilize C4 photosynthesis, a more efficient form of photosynthesis in hot, dry environments.^94^ C4 plants, such as *Salsola* and *Chenopodium*, can fix carbon more effectively under high temperatures and water stress than C3 plants, which are more common in cooler, wetter climates. This adaptation helps maintain productivity even under extreme conditions.^95^^,^^96^ In a survey of 104 desert species across China, 64% of vascular plants were identified as C_4_; notably, 58.7% of these were annuals and shrubs uniquely adapted for high-temperature, drought-prone environments.^97^ Further, studies on *S. laricifolia*, a woody shrub from Xinjiang’s arid zones, revealed a hybrid C_3_–C_4_ NADP-ME enzyme. When overexpressed in *Arabidopsis*, it conferred increased drought and salt resilience—highlighting evolutionary adaptations in desert lineages.^98^

Salt tolerance is another critical adaptation for species in regions with high soil salinity. In the Sahara Desert, for example, *Z. album* has specialized mechanisms for tolerating high salt concentrations in the soil. This allows it to thrive in environments where other species would struggle, thereby maintaining productivity and contributing to nutrient cycling in saline soils.^99^ Research shows that *Z. coccineum* seed germinates better under 100–200 mM NaCl, aided by larger seed size and increased leaf succulence to buffer salt stress.^100^ Anatomical analyses of Egyptian Z. album reveal thick cuticles, succulent leaves, and reduced stomatal density—classic xerohalophyte traits that preserve water and prevent salt-induced damage.^101^ On top of that, physiological assays confirm high proline content and antioxidant enzyme activity in *Z. album* from Wadi Hagul, demonstrating its robust response to both drought and salinity.^102^ These adaptations underscore finely tuned survival strategies seen in desert flora across both China and Egypt—combining photosynthetic efficiency and stress resilience to thrive under extreme conditions.

Together, these findings indicate that stress tolerance in desert plants is not governed by a single trait but by coordinated trait syndromes shaped by evolutionary history. In China, productivity persistence relies heavily on physiological efficiency (e.g., C_4_ photosynthesis and salinity tolerance), whereas in Egypt, it is more strongly mediated by ecosystem engineering traits such as nitrogen fixation, canopy shading, and soil improvement.

### Role of functional groups in ecosystem productivity

#### Classification of functional groups in desert and semi-arid ecosystems

In desert and semi-arid ecosystems, plant species are classified into functional groups based on their shared ecological roles and functional traits (Table 3). These functional groups, rather than individual species, help define ecosystem productivity and resilience. Functional groups are typically categorized based on characteristics such as plant growth forms, photosynthetic pathways, water use strategies, nutrient acquisition, and reproductive strategies.^111^^,^^112^ Understanding these groups is essential for predicting ecosystem responses to climate change and assessing the potential for sustainable land management practices.

**Table 3:** Functional groups and their traits in desert ecosystems

Functional group	Key traits	Examples (Egypt)	Examples (China)	Ecological role	Adaptations to stress	Contribution to productivity	Reference
C3 plants	use Calvin cycle for photosynthesis, common in cooler, moister environments	*Avena sativa*, *Lolium perenne*, *Trifolium alexandrinum*, *Phalaris minor*	*Triticum aestivum*, *Hordeum vulgare*, *Leymus chinensis*, *Festuca arundinacea*	moderate productivity in cooler climates	less efficient in hot, dry conditions	low productivity in arid regions, but important in cooler, wetter microclimates	Elkhouly et al.,^54^ Lv et al.^103^
C4 plants	efficient photosynthesis in hot, dry conditions, reduce water loss	*Chenopodium quinoa*, *Amaranthus viridis*, *Echinochloa colona*, *Cynodon dactylon*	*Salsola kali*, *Setaria viridis*, *Panicum miliaceum*, *Chenopodium glaucum*	high productivity in hot, dry environments	efficient water use, heat tolerance	high productivity in arid regions, especially during warm seasons	Elkhouly et al.,^54^ Lv et al.^103^
CAM plants	open stomata at night to minimize water loss, common in extremely dry deserts	*Opuntia ficus-indica*, *Euphorbia abyssinica*, *Sansevieria ehrenbergii*, *Aloe vera*	*Agave americana* (introduced), *Kalanchoe daigremontiana*, *Sedum alfredii*, *Bryophyllum pinnatum*	water conservation, survival in extreme drought	nighttime CO_2_ fixation, water storage	low but consistent productivity in extremely dry conditions	Abdelghnai & Hussein,^104^ Liu et al.^105^
Perennials	deep-rooted, survive long droughts, stabilize soil	*Acacia tortilis*, *Tamarix nilotica*, *Ziziphus spina-christi*, *Capparis decidua*	*Haloxylon ammodendron*, *Nitraria tangutorum*, *Calligonum mongolicum*, *Caragana korshinskii*	long-term soil stabilization, carbon sequestration	deep roots, drought resistance	high long-term productivity and ecosystem stability	Abdelghnai & Hussein,^104^ Liu et al.^106^
Annuals	complete life cycle in one season, rely on sporadic rainfall	*Chenopodium murale*, *Amaranthus albus*, *Anastatica hierochuntica*, *Plantago ovata*	*Salsola affinis*, *Suaeda glauca*, *Bassia dasyphylla*, *Chenopodium album*	rapid growth during wet periods, contribute to short-term productivity	ephemeral life cycle, rapid reproduction	high productivity during wet periods, but low during droughts	Amro et al.,^83^ Fei et al.^107^
Xerophytes	drought-resistant, water-conserving adaptations (e.g., thick cuticles)	*Zygophyllum coccineum*, *Anabasis articulata*, *Haloxylon salicornicum*, *Pergularia tomentosa*	*Reaumuria trigyna*, *Zygophyllum xanthoxylon*, *Nitraria sibirica*, *Kalidium foliatum*	soil stabilization, water retention, nutrient cycling	thick cuticles, reduced leaf surface area	moderate productivity, but critical for soil and water conservation	Elkhouly et al.,^54^ Zuo et al.^108^
Nitrogen-fixers	enhance soil fertility by fixing atmospheric nitrogen	*Acacia* spp., *Sesbania sesban*, *Medicago sativa*, *Prosopis juliflora*	*Alhagi camelorum*, *Sophora alopecuroides*, *Caragana microphylla*, *Lespedeza bicolor*	improve soil fertility, support other species	nitrogen fixation, deep roots	enhances productivity of surrounding plants, critical in nutrient-poor soils	Bidak et al.,^109^ Li et al.^110^

##### Growth Forms

One of the primary ways plants are classified in desert and semi-arid ecosystems is by their growth form. Perennials and annuals are the two primary groups in these regions, with perennials being dominant in many desert ecosystems due to their ability to survive long periods of drought. Perennial desert plants, such as *A. tortilis* in Egypt and *H. ammodendron* in China, possess deep root systems that enable them to access groundwater, playing a crucial role in maintaining ecosystem stability under arid conditions. In the Wadi Feiran basin of South Sinai, Egypt, Abd El-Wahab et al.^113^ examined the population structure of *A. tortilis* subsp. *raddiana* across a gradient of water availability. Their analysis of 289 trees across thirteen isolated populations revealed strong positive correlations between water availability indicators (e.g., catchment area and lineament density) and tree traits such as height, crown diameter, and trunk circumference. These findings indicate that variations in water access significantly influence the growth and density of *A. tortilis* populations. Similarly, a study by Wu et al.^114^ in the Gurbantunggut Desert of China investigated the drought response of two *Haloxylon* species in relation to groundwater depth. The researchers found that *H. ammodendron* exhibited a marked decline in growth as groundwater levels dropped, whereas *H. persicum* remained relatively unaffected. During periods of extreme drought, both species shifted to deeper water sources; however, they differed in their strategies. *H. ammodendron* derived 56–100% of its water from layers near the groundwater table, while *H. persicum* sourced 64–100% from deeper soil layers. Despite this adaptive shift in water uptake, the physiological performance of *H. ammodendron* was still compromised under declining groundwater conditions, suggesting that continued decreases in water availability could severely impact its survival. Together, these studies underscore the critical dependence of deep-rooted perennials on groundwater and highlight their vulnerability to increasing drought severity and groundwater depletion—factors that could destabilize desert ecosystems over time.

Annuals in desert ecosystems are adapted to complete their life cycles within a single growing season, often during brief rainfall events. Although they may boost productivity in wet years, their long-term ecological impact is limited due to reliance on sporadic precipitation.^115^ In the Gurbantünggüt Desert, China, Liu et al.^116^ studied four annual *Salsola* species and found drastic interannual population fluctuations, with 41.6–100% seedling mortality in spring. Fruit heteromorphism varied within and among species but was not directly linked to germination timing. Instead, germination heterochrony was driven by short seed longevity (one year) and rainfall patterns. Germination peaked at 0°C–15 °C and 20% soil moisture, highlighting sensitivity to environmental conditions. Similarly, Mahmoud et al.^117^ reported that *Chinopodium* (an annual plant) growth and yield in Egypt’s Nile Delta were influenced by water salinity. Freshwater irrigation (EC = 0.65 dS m^−1^) produced the highest biomass and seed yields, while increased salinity reduced yields but increased 1000-seed weight. Powdery mildew appeared around 53 days after sowing, likely due to high relative humidity. Biomass was significantly affected by environmental factors, with seed yield particularly sensitive to water salinity.

This contrast highlights a key ecological principle: In deserts where productivity is maintained by long-lived perennials (e.g., *Haloxylon*, *Acacia*), ecosystem stability depends disproportionately on groundwater and adult survival, whereas systems dominated by annual pulses are more sensitive to interannual rainfall variability but recover rapidly following favorable conditions.

##### Photosynthetic Pathways

Another important classification of plant species in desert and semi-arid ecosystems is based on the photosynthetic pathway. Plants in these ecosystems typically utilize one of the three photosynthetic pathways: C3, C4, and CAM (crassulacean acid metabolism) (Table 3). These pathways determine how plants process carbon dioxide and manage water use, which is crucial for productivity under stress.^118^

***C3 Plants:*** These plants use the Calvin cycle to fix carbon and are more common in cooler and moister environments.^26^ However, some C3 grasses in semi-arid regions may still thrive under moderate drought conditions^26^ (Table 3). In northern desert regions of China, Lv et al.^103^ found that long-term warming and increased precipitation significantly boosted C3 plant photosynthesis, with net rates rising by up to 159.5% under the highest treatment levels. Redundancy analysis showed that soil water content was the strongest predictor of photosynthetic performance (explaining 48% of variation), followed by soil-available nitrogen (19.6%). These results highlight water availability as a key driver of C3 plant function under climate change in arid and semi-arid ecosystems.

***C4 Plants:*** C4 photosynthesis is more efficient in hot and dry conditions, making C4 plants dominant in arid regions.^26^ C4 plants, such as *S. collina* and *Chenopodium*, concentrate carbon dioxide in specialized cells, which reduces water loss and increases photosynthetic efficiency^94^ (Table 3). Y. Liu^119^ explained that the C4 system in desert plants features spatial separation of carbon fixation between mesophyll and bundle sheath cells. Compared to the single-cell C4 species *Suaeda aralocaspica*, *S. ferganica* shows both similarities in structural and genetic enhancement and distinct physiological traits. These findings suggest diversity in C4 photosynthetic mechanisms and provide insights into the evolutionary trajectory of the *Salsola* genus.

***CAM Plants:*** CAM plants, such as agave and cactus species, take in carbon dioxide at night, which minimizes water loss during the hot daytime temperatures.^120^^,^^121^ These plants are particularly common in extremely dry deserts, such as the Sahara and the Arabian deserts, and are important for ecosystem productivity in areas with limited water availability^122^ (Table 3). Nobel^123^ mentioned that *A. deserti*, shows a rapid stomatal response to rainfall due to its shallow roots and succulent leaves, enabling gas exchange even after the soil dries. Cool nighttime temperatures are critical for minimizing water loss and optimizing CO_2_ uptake. Its low transpiration ratio highlights exceptional water-use efficiency, essential for survival in arid desert conditions.

##### Water Use Strategies

Plants are also classified based on their water use strategies, which include drought resistance, water storage, and water use efficiency.^124^ Xerophytes, such as *Zygophyllum* spp. and *R. trigyna*, have evolved specialized structures such as thick cuticles, reduced leaves, and deep root systems that enable them to conserve water and endure prolonged dry periods^32^^,^^125^ revealed critical anatomical adaptations in native desert species such as *Z. album, A. articulata, S. tetrandra,* and *F. indica* from North Sinai, Egypt. Features such as thick cuticles, dual-surface palisade layers, and water storage cells in *Z. album* leaves enhance drought resilience. Stem and root adaptations, including sclerenchymatous fibers and increased xylem vessels in *F. indica* and *Z. album*, support structural integrity and water uptake.

Hydrophytes, though less common in arid environments, may be present around oasis areas or regions with shallow groundwater, where water availability is higher. These plants play a critical role in stabilizing local microhabitats and supporting biodiversity. Imin et al.^126^ highlighted the critical role of groundwater availability (2.1–4.3 m) in maintaining the ecological function and coexistence of *P. euphratica* and *T. ramosissima* in the Daryaboyi Oasis of China. Big-sized plants and *T. ramosissima* exhibit greater salt tolerance and adaptability under groundwater stress compared to *P. euphratica*. As groundwater depth increases, *P. euphratica* prioritizes hydraulic efficiency by reducing growth, while *T. ramosissima* enhances water use efficiency before declining. These insights support targeted ecological restoration and sustainable groundwater management in arid inland river basins.

### Functional traits influencing productivity under climate stress in China and Egypt

In desert and semi-arid ecosystems, functional traits of plants determine their ability to withstand extreme climatic stressors such as high temperatures, water scarcity, and soil nutrient limitations. These traits can enhance or limit a plant’s contribution to ecosystem productivity under changing climate conditions.

#### Drought Resistance and Water Conservation

One of the most important functional traits for plant survival and productivity in arid and semi-arid environments is drought resistance.^127^ Plants such as *H. ammodendron* and *A. tortilis* possess deep root systems that allow them to access water from deeper soil layers, helping them survive prolonged dry spells.^113^^,^^114^ In South Sinai, *A. tortilis* growth traits positively correlated with water availability, indicating its sensitivity to hydrological conditions.^113^ In China’s Gurbantunggut Desert, *H. ammodendron* showed growth decline with groundwater drop, while *H. persicum* remained stable. Both species adapted by shifting water uptake to deeper layers, yet *H. ammodendron* remained physiologically vulnerable. These findings underscore deep-rooted perennials’ reliance on groundwater and their risk under increasing drought.^114^ Moreover, these species often have leaves with specialized structures, such as waxy coatings or reduced surface areas, to minimize water loss through transpiration. Hu et al.^128^ conducted a study in Aibihu Wetland National Nature Reserve in Jinghe County, Xinjiang Uygur Autonomous Region—a northwest inland region of China- and demonstrated that *H. ammodendron* exhibited adaptive plasticity in leaf morphology to cope with variable soil conditions. *H. ammodendron* adopted a high specific leaf area under favorable soil conditions to maximize carbon assimilation.

#### Heat Tolerance and Temperature Regulation

Tolerance to high temperatures is another crucial functional trait in desert ecosystems.^38^ Many desert plants, such as *Zygophyllum* spp. and *R. trigyna*, have evolved physiological mechanisms to minimize heat stress, including leaf morphologies that reflect sunlight or store water, which helps them endure extreme heat.^129^ Bai et al.^130^ mentioned that *Z. xanthoxylum* (a typical desert plant in China's deserts) demonstrates a distinct thermotolerance mechanism that enhances its survival in desert environments. Under moderate to high temperatures (40 °C), the plant shows improved photosynthetic capacity and growth performance. However, exposure to severe heat stress (45 °C) results in reduced photosynthesis and growth inhibition. Transcriptomic analysis revealed significant upregulation of heat shock proteins (HSPs), heat shock transcription factors (HSFs), and photosystem-related genes. Additionally, the downregulation of chlorophyll catabolism genes contributes to maintaining chlorophyll levels under heat. These coordinated physiological and molecular responses underpin *Z. xanthoxylum’s* adaptation to extreme thermal conditions.

Date palm (*Phoenix dactylifera*), a keystone species in Egyptian oases such as those in the Sinai and Sahara regions, demonstrates remarkable heat tolerance. A recent study found that several HSPs, including HSP17.6, HSP22, HSP23.6, and HSP26.5, play vital roles in protecting the plant during high-temperature stress. When these HSPs were expressed in *A. thaliana*, they significantly improved photosynthetic efficiency, as indicated by enhanced Fv/Fm ratios under heat stress conditions of approximately 37 °C.^131^ These findings suggest that date palms rely on HSP-mediated mechanisms to maintain leaf photosynthetic function during extreme heat events—a critical adaptation for survival in arid environments. *Anastatica hierochuntica*, commonly known as the resurrection plant and native to the Sahara and Sinai deserts, serves as a model desert annual. Transcriptomic comparisons between *A. hierochuntica* and *Arabidopsis* reveal that this desert species possesses a highly flexible and rapid heat stress response. It swiftly activates genes involved in stomatal regulation, DNA repair, and protective cellular functions in response to extreme diurnal temperature fluctuations—ranging from −3.6 °C at night to 46.8 °C during the day.^132^ This molecular plasticity enables *A. hierochuntica* to thrive in hyper-variable desert climates and highlights its potential as a model for studying stress resilience in arid environments.

#### Nutrient acquisition strategies

Desert plants have evolved specialized adaptations to acquire nutrients in soils typically deficient in nitrogen, phosphorus, and other essential elements.^133^ Nitrogen-fixing species such as *Acacia* and *Alhagi* play a pivotal role in these nutrient-poor environments by converting atmospheric nitrogen into forms accessible to plants. This process not only boosts the growth of these nitrogen-fixing species but also enriches the surrounding soil, thereby enhancing the productivity of neighboring vegetation.^134^ In Egypt, Hassan and Hamdy^135^ provided an updated checklist of *Acacia* species, with a focus on 24 exotic taxa documented as either living specimens or herbarium records. More recently, Tariq et al.^136^ published a comprehensive review on *A. sparsifolia* in Chinese desert ecosystems, emphasizing its ecological role in nitrogen fixation and its significance in combating desertification under extreme arid conditions. The uneven distribution of nitrogen-fixing taxa between regions suggests that Egyptian deserts may sustain higher baseline soil fertility and facilitation-driven productivity than Chinese cold deserts, where nutrient limitation is often more severe and recovery following disturbance is slower. This difference has direct implications for restoration success and carbon sequestration potential.

#### Phenological adaptations

The timing of key life-history events—such as germination, flowering, and fruiting—is a crucial functional trait that shapes ecosystem productivity.^137^ In arid environments, species such as *Salsola* and *Chenopodium* have evolved to respond rapidly to brief wet periods, allowing them to complete their reproductive cycles before the onset of drought. These phenological adaptations enable plants to make optimal use of limited water availability and enhance overall ecosystem function.^138^ A study conducted in the Junggar Basin, particularly the central Gurbantünggüt Desert in China, found that *Salsola* species exhibit germination heterochrony as a key survival strategy. Although fruit heteromorphism was observed, germination timing was mainly influenced by temperature, moisture, and short seed longevity. Peak germination (50%) occurred at 0°C–15 °C with 20% soil moisture, and seed viability lasted only one year. This adaptive strategy allows *Salsola* to mitigate the risks posed by unpredictable rainfall and high seedling mortality in desert environments.^116^ Moreover, a recent study on *P. euphratica* in central Taklamakan oases used satellite data and field observations (2004–2023). Results show that start-of-season (SOS) and end-of-season (EOS) phenology shifted depending on groundwater depth: Regions with shallow groundwater (<8 m) showed earlier SOS and extended EOS under warming, while deeper groundwater saw delayed SOS and EOS due to moisture constraints.^139^ Similarly, research on several spring annuals and shrubs in South Sinai—including *Primula boveana*—shows germination tied to winter rain and cold stratification, with flowering and seed set completed within 60–90 days.^140^

### Interactions between functional groups and ecosystem stability

The interactions among different functional plant groups in desert and semi-arid ecosystems are vital for sustaining ecosystem stability and productivity. These interactions occur across several ecological levels, including competition, facilitation, and mutualistic relationships. The balance of these interactions influences not only species survival but also the broader ecological functions of these harsh environments.^141^^,^^142^

Facilitation is a key mechanism in desert ecosystems, where one species creates conditions that benefit others.^143^ In Egypt’s Sinai Desert, *A. tortilis*—a deep-rooted leguminous tree—fixes atmospheric nitrogen through symbiotic relationships with rhizobia. This process enriches nitrogen-poor soils, supporting the growth of surrounding shrubs and grasses.^113^ In Chinese deserts, such as the Taklamakan and Gurbantünggüt, *A. sparsifolia*, another nitrogen-fixing legume, contributes to soil fertility and provides shelter and improved microclimate conditions for smaller desert species.^136^ Similarly, deep-rooted perennials such as *H. ammodendron* can draw water from deep soil layers and improve surface moisture conditions, thereby facilitating the establishment of shallow-rooted species during short wet periods.^144^

Despite the importance of facilitation, competition remains a strong force in desert plant communities, especially where water and nutrients are extremely limited. Species compete based on rooting depth, photosynthetic pathways, and temporal resource use.^145^ For example, in China’s arid zones, C4 species such as *S. collina* may outcompete C3 plants such as *A. desertorum* during periods of intense heat and light due to their higher water-use efficiency.^146^ In Egypt, shallow-rooted annuals often compete for surface moisture during brief rainfall events, while deep-rooted perennials such as *T. nilotica* maintain access to groundwater, giving them a consistent advantage during prolonged droughts.^147^ However, resource partitioning—where species specialize in different niches—often reduces the intensity of competition, allowing coexistence of multiple functional types.^148^

Species interactions strongly influence community structure and functional group dominance.^149^ In both Egyptian and Chinese deserts, succulents and shrubs play a significant role in moderating microhabitats. For instance, *Z. xanthoxylum* in China’s desert steppes can provide shade and reduce soil evaporation, creating conditions conducive to the germination of annual grasses such as *C. album*.^150^ Similarly, in Egypt’s Western Desert, shrubs such as *Z. spinosa* can protect the seedlings of more delicate species by buffering extreme temperatures and reducing wind erosion.^151^ These positive interactions enhance species diversity and productivity, making the ecosystem more resilient to environmental fluctuations. Collectively, these interaction networks indicate that ecosystem stability in Egyptian deserts is strongly facilitation-dependent and vulnerable to keystone species loss, whereas Chinese desert ecosystems rely more on functional redundancy among shrub species, potentially conferring greater resistance to species turnover but lower capacity for rapid nutrient enhancement.

## Impact of extreme climate events on plant productivity

**Figure 4:**
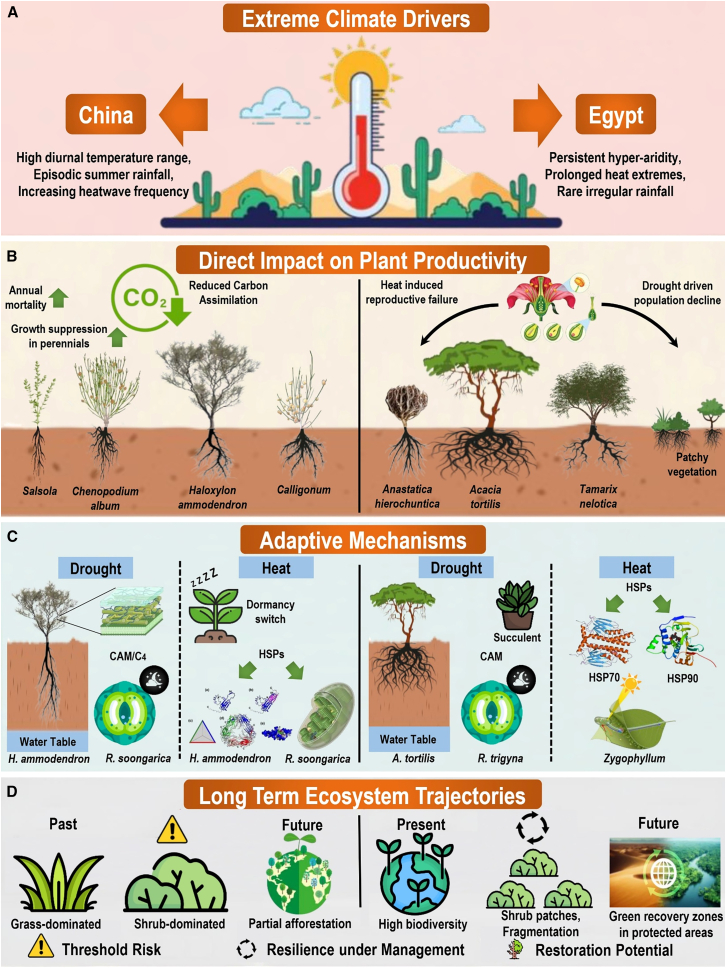
Impacts of extreme climate events on plant productivity and ecosystem resilience in arid regions of China and Egypt This conceptual figure synthesizes how intensifying droughts and heatwaves regulate plant productivity through direct physiological stress, species-specific responses, adaptive mechanisms, and long-term ecosystem trajectories in the deserts of China and Egypt. (A) Illustrates the dominant climatic drivers shared by both regions—prolonged drought, extreme heat (>40°C–45 °C), high solar radiation, and declining or highly variable precipitation—while highlighting regional climatic contrasts, including strong diurnal temperature fluctuations and episodic summer rainfall in China versus persistent hyper-aridity and prolonged heat extremes in Egypt. (B) Depicts the immediate impacts of these stressors on plant communities and productivity. In China, drought and heat disproportionately reduce the survival and productivity of shallow-rooted annuals (e.g., *Salsola*, Quinoa/*Chenopodium*), while deep-rooted perennials such as *Haloxylon ammodendron* and *Calligonum* spp. exhibit suppressed photosynthesis, reduced growth, and partial canopy loss during extended stress. In Egypt, extreme heat and water scarcity cause leaf senescence, reduced flowering and seed set, and declining vegetation cover in annuals (e.g., *Anastatica hierochuntica*), while dominant perennials such as *Acacia tortilis* and *Tamarix nilotica* show reduced growth and reproductive output under persistent stress. (C) Summarizes key adaptive mechanisms that enable plant persistence under drought and heat. In China, adaptive strategies include deep or flexible rooting systems accessing groundwater, reduced leaf area and waxy cuticles, high water-use efficiency (C4 and CAM pathways), stress-induced dormancy, and the activation of heat shock proteins and chloroplast protection mechanisms. In Egypt, survival strategies emphasize ultra-deep root systems (>30 m), small or thick leaves, reflective surfaces, CAM photosynthesis, sunken stomata, and strong molecular defenses involving heat shock proteins (e.g., HSP70, HSP90, sHSPs), dehydrins, and LEA proteins that stabilize cellular structures under extreme thermal and drought stress. (D) Integrates these processes over time to illustrate long-term ecosystem trajectories. In China, increasing frequency and severity of droughts and heatwaves promote shrub dominance, reduced grass cover, and heightened desertification risk, with partial recovery possible through afforestation and restoration initiatives. In Egypt, prolonged aridification drives biodiversity loss, habitat fragmentation, and localized resilience largely confined to oases and protected areas. Collectively, the figure highlights how extreme climate events reshape productivity, community structure, and resilience in desert ecosystems, while emphasizing the critical role of species traits, functional strategies, and management interventions in buffering future climate impacts.

Extreme climate events—particularly prolonged droughts and heatwaves—are increasingly dominant drivers of plant productivity and community dynamics in arid and semi-arid ecosystems. In the deserts of China and Egypt, these episodic but intensifying stresses disrupt physiological processes, alter species interactions, and amplify existing resource limitations (Table 4). This section examines how drought and heat affect plant communities, the adaptive mechanisms that enable key species and functional groups to persist, and the long-term implications for ecosystem resilience under ongoing climate change (Figure 4).

**Table 4:** Impacts of climate change on Plant productivity in China and Egypt

Impact	China	Egypt	Similarities	Differences	Implications for ecosystem productivity	Reference
Temperature rise	shifts in species composition, cold-adapted species decline	increased evaporation, reduced water availability	both face increased heat stress and species shifts	China’s cold-adapted species are more affected; Egypt’s heat-tolerant species thrive	reduced productivity in China due to cold-adapted species decline; Egypt faces increased water stress	Chen et al.,^55^ Gado & El-Agha^152^
Drought	reduced productivity, especially for annual species	expansion of desert areas, loss of shallow-rooted species	both face water scarcity and reduced productivity	China’s droughts are more seasonal; Egypt’s are more prolonged	seasonal droughts in China reduce productivity; Egypt’s prolonged droughts lead to desertification	Elbeih,^153^ Liu et al.^154^
Heat stress	reduced photosynthetic activity, protein denaturation	stunted growth, reduced reproductive success in dominant species	both experience reduced growth and productivity under heat stress	China’s heat stress is seasonal; Egypt’s is year-round	reduced photosynthesis in both regions, but Egypt’s year-round heat stress is more severe	Bedair et al.,^17^ Yu et al.^155^
Species shifts	drought-tolerant shrubs become more dominant	drought-tolerant species thrive, but overall productivity may decline	both experience shifts toward drought-tolerant species	China’s shifts are toward cold-tolerant species; Egypt’s toward heat-tolerant	productivity may stabilize in China with cold-tolerant species; Egypt’s productivity may decline overall	Gou et al.,^156^ El-Ghani et al.^157^
Extreme events	increased frequency of droughts and heatwaves, reduced resilience	prolonged droughts, heatwaves, and reduced ecosystem recovery	both face increased frequency of extreme events	China’s events are more seasonal; Egypt’s are more consistent	reduced resilience in both regions, but China’s ecosystems may recover faster due to seasonal variability	Wang et al.,^158^ Tu et al.^159^

### Drought and heat stress effects on plant communities

#### Impact of drought stress on plants

Drought is one of the most critical limiting factors for plant growth in desert ecosystems. Water availability is essential for physiological processes such as photosynthesis, nutrient uptake, and cell expansion.^160^ During prolonged dry spells or periods of low rainfall, plants experience water stress, which impairs their ability to maintain turgor pressure and perform essential metabolic functions.^161^ To conserve water under drought conditions, many desert plants reduce transpiration rates, leading to slower growth and reduced productivity.^162^ In severe cases, prolonged drought stress causes leaf shedding, diminished flowering and fruiting, and even mortality, particularly among annual species and shallow-rooted plants.^163^

In China’s Taklamakan and Gurbantünggüt Deserts, annual species such as *S. collina*, *S. ferganica,* and *C. album* depend heavily on sporadic rainfall for germination and development, making them especially vulnerable to drought.^164^^,^^165^^,^^166^^,^^167^ Conversely, perennial species such as *H. ammodendron* and *C. mongolicum*, with their extensive root systems, are better equipped to access deep soil moisture and survive longer dry periods.^168^^,^^169^ However, even these drought-tolerant species can exhibit reduced photosynthetic activity and slowed growth during extended droughts.^170^ Similarly, in Egypt’s arid regions, including the Sinai and Western Deserts, annuals such as *Anastatica hierochuntica* and *F. arabica* suffer rapid population declines during drought years due to their short life cycles and limited root depth.^54^ In contrast, perennials such as *A. tortilis* and *T. nilotica* demonstrate higher drought resilience thanks to their deep-rooting systems and morphological adaptations such as small, thick leaves that minimize water loss. Despite their resilience, persistent drought can still reduce their growth rates and reproductive success, affecting the overall productivity of desert ecosystems.^171^^,^^172^ These patterns underscore the importance of root architecture, life history traits, and physiological adaptations in determining plant survival and ecosystem stability under water-limited conditions (Figure 4B). These parallel responses reveal that drought sensitivity is structured not only by life history but also by evolutionary background: Cold-desert shrubs in China tend to prioritize hydraulic safety and dormancy, whereas hot-desert trees in Egypt emphasize sustained carbon gain and hydraulic access, leading to different resilience trajectories under prolonged drought.

#### Impact of heat stress on plants

Heat stress intensifies the adverse effects of drought, especially in desert regions where summer temperatures often exceed 40 °C.^173^ Under extreme heat, key physiological processes in plants are disrupted—photosynthetic enzymes become inactivated, protein structures denature, and cellular membranes are damaged.^174^ Elevated temperatures also accelerate transpiration, increasing water loss at a time when water availability is already limited. This creates a compounded stress scenario where plants are unable to balance water loss with uptake, resulting in dehydration, impaired growth, and reduced reproductive output.^175^

In Egypt’s Eastern and Western Deserts, frequent summer heatwaves affect native flora such as *T. nilotica* and *Calligonum* spp, both of which are dominant species in sandy and rocky habitats. These species may exhibit leaf senescence, suppressed flowering, and reduced seed set under extreme temperatures.^171^^,^^172^ Similarly, in the deserts of northwestern China, including the Junggar and Tarim Basins, dominant species such as *A. aphylla* and *E. przewalskii* often experience reduced chlorophyll content and weakened carbon assimilation under high heat conditions.^176^ In both regions, the combination of heat and drought stress significantly alters competitive dynamics, decreases vegetation cover, and contributes to shifts in plant community structure—ultimately reducing biodiversity and impairing ecosystem functioning. Thus, while both regions experience productivity declines under heat stress, Egyptian deserts—already near upper thermal limits—may face sharper physiological thresholds, whereas Chinese deserts may experience more pronounced shifts in species composition as warming relaxes cold constraints but intensifies water limitation.

### Adaptive mechanisms of key plant species and functional groups

#### Drought adaptations

Many desert plants have evolved specialized structural and physiological adaptations to survive extreme aridity. One of the most important traits is the development of deep or extensive root systems, enabling access to groundwater far below the surface. For example, *A. raddiana* in Egypt and *H. ammodendron* in China can extend their roots several meters deep to tap into subsurface water reserves, allowing them to thrive in regions with minimal rainfall.^54^^,^^114^ In addition to root adaptations, leaf modifications are also critical for reducing water loss. Species such as *Z. coccineum* in Egypt and *R. soongarica* in China possess small, waxy, or reduced leaves that limit transpiration by minimizing surface area and enhancing water retention.^102^^,^^177^ Some desert plants, including various cacti and Agave species, take water conservation further by replacing leaves with spines and storing water in their fleshy tissues.^178^ These succulents also utilize CAM photosynthesis, a water-efficient strategy that allows them to open stomata at night when temperatures are lower, thereby reducing evaporative water loss.^179^ Together, these adaptations enable plants in both Chinese and Egyptian deserts to survive prolonged droughts and maintain ecological function in some of the world’s harshest environments (Figure 4C).

#### Heat stress adaptations

Plants thriving in hot desert environments have evolved a sophisticated suite of physiological and cellular adaptations to cope with extreme heat and arid conditions. Notably, species such as *A. tortilis* and *R. trigyna* produce heat-resistant proteins that play essential roles in maintaining cellular function under thermal stress.^180^ In *A. tortilis*, key HSPs include HSP70, which assists in protein folding and prevents aggregation; HSP90, which stabilizes and refolds denatured proteins while participating in stress signal transduction; and small HSPs (sHSPs), which act as molecular chaperones rapidly expressed under heat conditions.^181^

Similarly, *R. trigyna* expresses HSP70 and HSP101, both of which are upregulated during heat and combined drought-heat stress, aiding in protein stabilization.^182^ Additionally, this species produces dehydrins and LEA (Late Embryogenesis Abundant) proteins, which, although not classical HSPs, function comparably by protecting proteins and membranes during desiccation and high temperatures, along with sHSPs that safeguard cytoplasmic and chloroplast proteins.^183^ Beyond biochemical defenses, both species exhibit specialized cellular structures to tolerate heat. In *A. tortilis*, features include a thick cuticle and epidermal layers that reduce water loss and reflect sunlight, trichomes that increase leaf reflectance and create a humid microclimate, heat-stable chloroplast membranes that preserve photosynthetic function, vacuoles storing osmoprotectants such as proline and glycine betaine, and sunken stomata that close rapidly to minimize transpiration.^184^

In *R. trigyna*, heat tolerance is further supported by thickened cell walls and xeromorphic leaf architecture to limit heat and water loss, chloroplasts containing plastoglobules that protect thylakoid membranes, cytoplasmic heat shock granules that isolate damaged proteins, and heat-stable mitochondria and peroxisomes that maintain metabolic function while mitigating oxidative stress.^185^ Moreover, crystalline protein arrays in the cytosol may help stabilize enzymes during extreme heat.^186^ Collectively, these molecular and structural adaptations provide crucial protection against heat-induced damage at the molecular level, enabling them to endure soaring temperatures that often exceed 45°C. Additionally, certain desert plants, such as *Zygophyllum* species, have developed reflective leaf surfaces that effectively deflect intense sunlight, significantly reducing the amount of heat absorbed by their tissues and thereby preventing overheating and cellular injury.^187^

The combined stress of drought and heat presents an even greater challenge, prompting plants such as *Salsola* and *R. trigyna* to strategically downregulate metabolic processes such as photosynthesis to conserve precious water and energy resources.^164^^,^^188^ Some desert-adapted species, including *H. ammodendron*, employ dormancy as a survival strategy, entering a state of metabolic inactivity during the harshest periods and resuming growth and physiological activity only when environmental conditions become favorable again.^189^ These complex and multi-faceted adaptations highlight the incredible resilience and resourcefulness of desert plants in coping with one of the most extreme habitats in China and Egypt (Figure 4C).

### Long-term trends and resilience of ecosystems in China and Egypt

Over the long term, both China and Egypt have experienced shifts in their desert ecosystems as a result of climate change, human activity, and the natural variability of environmental stressors such as drought and heat. These long-term changes have implications for the resilience of these ecosystems and the ability of plant communities to maintain productivity (Figure 4D).

In China, the desertification process in the Taklamakan Desert and other arid regions has been accelerated by factors such as overgrazing, deforestation, and excessive water extraction for agriculture.^190^ Climate change has also exacerbated water scarcity, resulting in shifts in plant species composition. While some species, such as *H. ammodendron*, have shown resilience to these changes due to their deep-rooted nature, other species have struggled to cope with the intensifying stress.^191^ Moreover, the increased frequency and severity of heatwaves and droughts have made it harder for these ecosystems to recover from disturbance events. Similarly, in Egypt, desertification and land degradation, largely driven by human activities such as overgrazing and unsustainable agricultural practices, have significantly altered the Sahara and the Eastern Desert’s ecological stability.^192^ *A. tortilis* and other dominant species have shown some resilience due to their adaptations to heat and drought, but the loss of biodiversity, along with increased temperatures and more frequent droughts, threatens the long-term sustainability of desert ecosystems.^86^^,^^113^

Despite these challenges, desert ecosystems in both countries have shown remarkable resilience. Long-term monitoring and restoration efforts, such as afforestation and reforestation programs, have helped stabilize degraded lands and improve ecosystem functions.^193^ In China, for instance, the introduction of drought-tolerant species such as *Caragana* and *Atriplex* in afforestation projects has helped restore degraded areas and reduce soil erosion.^194^^,^^195^ Similarly, in Egypt, the establishment of protected areas and sustainable land management practices has contributed to the recovery of key plant species such as *A. tortilis*, improving ecosystem resilience.^196^

Overall, the China-Egypt comparison reveals that resilience in desert ecosystems emerges from different dominant mechanisms: structural persistence and redundancy in cold deserts versus biogeochemical facilitation and keystone dependence in hot deserts. Recognizing these divergent resilience pathways is critical for predicting ecosystem responses to climate extremes and for designing region-specific restoration and management strategies.

#### Human-induced changes and management strategies

**Figure 5:**
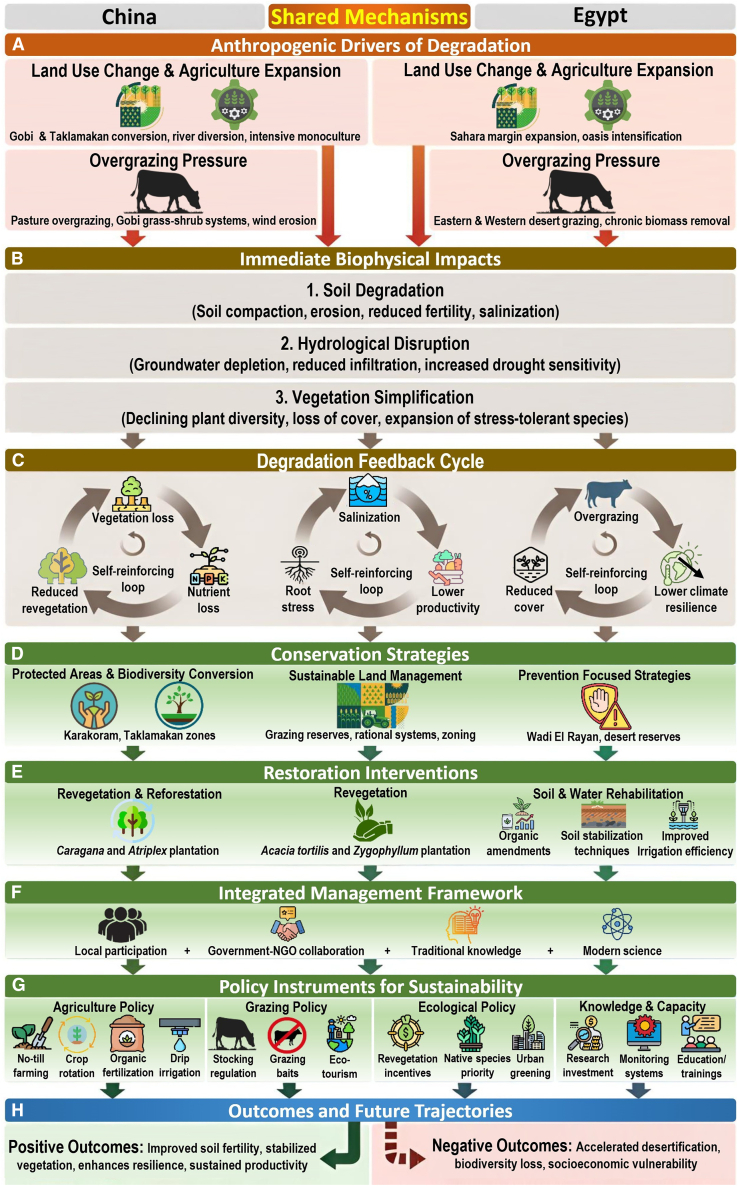
Human-induced drivers of degradation and integrated management strategies sustaining desert ecosystem productivity in China and Egypt This conceptual figure synthesizes the pathways through which anthropogenic pressures reshape arid and semi-arid ecosystems and illustrates conservation, restoration, and policy interventions that can mitigate degradation and enhance long-term productivity. (A) Depicts the major human drivers, highlighting land-use change, agricultural expansion, and overgrazing in both regions. In China, conversion of the Gobi and Taklamakan desert margins to irrigated cropland, river diversion, intensive monoculture, and pastoral overgrazing exert strong pressure on soils and vegetation. In Egypt, long-term agricultural expansion into the Sahara, large-scale irrigation schemes, and intensive Bedouin grazing in the Eastern and Western Deserts similarly intensify land degradation. (B) Illustrates the immediate biophysical impacts of these drivers, including soil compaction, erosion, and salinization, depletion of groundwater resources, reduced soil water-holding capacity, and simplification of plant communities through the loss of native species and functional diversity. (C) Emphasizes self-reinforcing degradation feedbacks, where vegetation loss increases soil exposure, erosion, and nutrient depletion, further constraining plant regeneration and accelerating desertification. (D) summarizes conservation approaches focused on preventing further degradation, including the establishment of protected areas and biodiversity conservation zones (e.g., Karakoram and Taklamakan reserves in China; Wadi El Rayan and other desert protected areas in Egypt) and the adoption of sustainable land-management practices such as grazing reserves, rotational grazing, and land-use zoning. (E) Presents restoration approaches aimed at reversing degradation, including revegetation and reforestation with drought-tolerant native species (e.g., *Caragana* and *Atriplex* in China; *Acacia tortilis* and *Zygophyllum* spp. in Egypt), soil stabilization, organic amendments to improve fertility, and improved water-management practices. (F) Integrates conservation and restoration within a socio-ecological framework, highlighting the importance of collaboration among local communities, governments, and non-governmental organizations to ensure economically viable and socially acceptable management outcomes. (G) Outlines policy instruments that support sustainable ecosystem productivity, including incentives for sustainable agriculture (no-till farming, crop rotation, organic fertilization, drip irrigation, and rainwater harvesting), strengthened grazing management and alternative livelihoods, reforestation and revegetation policies prioritizing native species, and investment in research, monitoring, and education. (H) Illustrates projected outcomes and future trajectories, contrasting positive pathways—improved soil fertility, stabilized vegetation cover, enhanced biodiversity, and increased resilience to climatic extremes—with negative outcomes under inadequate management, such as accelerated desertification, biodiversity loss, and heightened socio-economic vulnerability. Collectively, the figure highlights how integrated, multi-level management strategies are essential for sustaining desert ecosystem productivity in China and Egypt under increasing human and environmental pressures.

Beyond climatic constraints, human activities have become major drivers of ecosystem degradation and productivity loss in arid and semi-arid regions. In China and Egypt, land-use change, agricultural expansion, and overgrazing have accelerated desertification, altered vegetation structure, and weakened ecosystem resilience. This section examines the impacts of these anthropogenic pressures (Table 5) and synthesizes conservation, restoration, and policy-based management strategies aimed at sustaining ecosystem productivity under increasing environmental and socio-economic pressures (Figure 5).

**Table 5:** Human-induced changes and management strategies

Aspect	China	Egypt	Similarities	Differences	Implications for ecosystem productivity	Reference
Land use changes	agricultural expansion, deforestation, overgrazing	agricultural expansion, overgrazing, water resource exploitation	both face significant land degradation due to human activities	China’s degradation is driven by colder climates; Egypt’s by extreme heat	reduced productivity due to habitat loss and soil degradation in both regions	Elsharkawy et al.,^197^ Wu et al.^198^
Desertification	accelerated by overgrazing and water extraction	expansion of desert areas due to unsustainable practices	both face desertification due to overexploitation	China’s desertification is more linked to cold climates; Egypt’s to heat	loss of productive land in both regions, but China’s desertification is more reversible	Ren et al.,^199^ Nour-Eldin et al.^200^
Conservation efforts	grain for green program, afforestation with drought-tolerant species	protected areas (e.g., Wadi El Rayan), revegetation programs	both have implemented conservation and restoration programs	China’s programs focus on cold-tolerant species; Egypt’s on heat-tolerant	improved soil stability and productivity in both regions, but long-term success depends on climate resilience	Shaltout & Bedair,^201^ Zhang et al.^202^
Restoration strategies	planting caragana, atriplex, soil stabilization, water management	reintroduction of *Acacia tortilis*, Zygophyllum, organic soil amendments	both focus on soil stabilization and water management	China’s strategies are more focused on cold regions; Egypt’s on hot regions	enhanced productivity and resilience in both regions, but challenges remain due to climate change	Elkhouly et al.,^54^ Zhou et al.^203^
Policy recommendations	sustainable agriculture, rotational grazing, reforestation	water-efficient irrigation, grazing management, community involvement	both emphasize sustainable land use and community involvement	China’s policies are more focused on cold climates; Egypt’s on heat and water	improved land management practices can enhance productivity, but require long-term commitment	Iwasaki et al.,^204^ Hu et al.^205^

### Land use changes, overgrazing, and agricultural expansion

#### Agricultural expansion

In both China and Egypt, agricultural expansion has been a major driver of land use changes.^70^^,^^206^ The conversion of natural desert and semi-arid landscapes into agricultural land often involves the draining of wetlands, diversion of rivers, and irrigation of arid lands.^207^ While agriculture provides economic opportunities, particularly for rural communities, it comes at a cost to the environment. The over-extraction of groundwater for irrigation, for instance, can lead to aquifer depletion, making these ecosystems more vulnerable to droughts.^208^ In Egypt, the Sahara Desert has been the target of agricultural projects for decades, with irrigated agriculture encroaching into once pristine areas.^153^ Similarly, in China, vast stretches of land in the Gobi Desert and Taklamakan Desert have been converted to farming, resulting in the degradation of the land and its natural vegetation.^209^ Irrigated agriculture, though intended to boost productivity, often leads to the accumulation of salts in the soil due to improper water management, which affects plant growth. This process, known as salinization, reduces the fertility of the land and diminishes ecosystem productivity over time.^210^ Additionally, monoculture practices, which are common in both countries, can reduce plant diversity and leave ecosystems more susceptible to pests and diseases, further harming ecosystem stability.^211^

#### Overgrazing

Overgrazing by livestock is another critical issue that affects desert and semi-arid ecosystems in China and Egypt.^212^^,^^213^ Livestock, such as goats, sheep, and cattle, exert high pressure on vegetation by consuming plant matter at unsustainable rates. This leads to soil compaction, erosion, and the depletion of plant cover, which in turn reduces the capacity of the land to retain water and nutrients.^214^ In Egypt, Bedouin communities have long practiced grazing in the deserts, and overgrazing is now a widespread problem in the Eastern Desert and Western Desert regions.^215^ Similarly, in China’s Gobi Desert, pastoral grazing has degraded vast stretches of land, leaving soils exposed to wind erosion.^216^ Overgrazing also reduces the abundance of native plant species, replacing them with more drought-tolerant, but less productive, species. This diminishes the overall primary productivity of the land and hinders its ability to recover from extreme climatic events such as droughts. The loss of vegetative cover leads to a vicious cycle where soil erosion reduces the fertility of the land, making it increasingly difficult for vegetation to regrow.^217^

### Conservation and restoration approaches

#### Conservation approaches

The focus of conservation efforts in desert ecosystems revolves around the protection of biodiversity, particularly the preservation of native species and natural habitats.^218^ Both China and Egypt have established protected areas and national parks in ecologically significant desert regions. For example, in China, the Karakoram Desert and parts of the Taklamakan Desert have been designated as conservation areas, helping protect vital plant and animal species from further exploitation.^80^^,^^219^ Similarly, in Egypt, the Wadi El Rayan Protected Area and other conservation zones have been set up to protect unique desert flora and fauna.^220^ Additionally, sustainable land management practices are a cornerstone of conservation efforts. This includes initiatives aimed at reducing overgrazing, such as the establishment of grazing reserves and the promotion of rotational grazing systems. In China, initiatives like the Grain for Green Program, which promotes land conversion from agricultural use back to forest or grassland, have been successful in combating desertification and restoring soil fertility.^221^

#### Restoration approaches

Restoration efforts aim to rehabilitate degraded land and bring it back to a productive state. In both China and Egypt, these efforts have focused on addressing soil erosion, improving water management, and reintroducing native vegetation. For example, China has invested heavily in desert reclamation projects that involve planting drought-resistant species such as *Caragana* and *Atriplex*. These species help stabilize the soil, prevent erosion, and provide important habitat for wildlife.^222^^,^^223^ In Egypt, restoration efforts have involved revegetation programs and the reintroduction of native shrubs such as *A. tortilis* and *Zygophyllum* spp. to prevent soil erosion and enhance ecosystem stability. The use of organic amendments, such as compost and manure, has also been promoted to improve soil fertility and enhance water retention capacity.^224^

#### Integrated approaches

An integrated approach that combines both conservation and restoration is essential for the long-term success of desert ecosystem management. In both China and Egypt, collaboration between local communities, governments, and non-governmental organizations (NGOs) is vital to the success of these programs.^225^ These collaborations focus on building local capacity for sustainable land use practices, ensuring that conservation and restoration efforts are economically viable for local communities.^226^^,^^227^

### Policy recommendations for sustaining ecosystem productivity

To ensure the long-term sustainability of desert ecosystems, policy recommendations are crucial for addressing the challenges posed by land use changes, overgrazing, and agricultural expansion. Several strategies are necessary to mitigate the impacts of these activities and promote ecosystem health.

(a) **Encouraging Sustainable Agricultural Practices**: Governments in both China and Egypt should incentivize sustainable farming techniques that minimize environmental degradation. Practices such as no-till farming, crop rotation, and the use of organic fertilizers can reduce soil erosion, enhance soil fertility, and decrease water consumption. In addition, promoting the use of drip irrigation systems and rainwater harvesting technologies can help conserve water in arid regions while ensuring agricultural productivity.^53^^,^^228^
(b) **Strengthening Grazing Management:** In areas where overgrazing is a major concern, policies should be implemented to regulate livestock numbers and enforce grazing bans in sensitive areas. Rotational grazing and the development of grazing reserves can help ensure that land is not overexploited. Additionally, promoting the use of alternative livelihoods for pastoral communities, such as ecotourism or sustainable livestock management, can reduce the pressure on desert ecosystems.^229^^,^^230^
(c) **Promoting Reforestation and Revegetation:** Governments should prioritize reforestation and revegetation efforts to restore degraded land. Policies should focus on selecting native plant species that are well adapted to local climate conditions and can thrive with minimal water inputs. Providing financial incentives for landowners to engage in revegetation activities and promoting the use of native plants for landscaping in urban areas can help increase the green cover in desert regions.^231^^,^^232^
(d) **Investing in Research and Education:** It is essential to invest in research to develop new technologies and methods for land management, water conservation, and soil restoration. Moreover, public awareness campaigns and education programs can help communities understand the importance of sustainable land use and the consequences of overexploitation.^233^^,^^234^

## Conclusion and future research directions

This review underscores the pivotal role of both dominant and lesser-known plant species and functional groups in maintaining ecosystem productivity in China’s and Egypt’s desert and semi-arid regions. Species such as *Atriplex, Caragana, Acacia*, and *Zygophyllum* contribute to soil stabilization, nutrient cycling, and water retention, while functional diversity enhances resilience to climatic stressors such as drought, heat, and salinization. However, despite these insights, significant knowledge gaps persist. The specific contributions of various plant species and functional groups across environmental gradients remain inadequately understood, particularly regarding their interactions and collective impact on nutrient cycling and carbon sequestration under climatic stressors. Moreover, the roles of rare or subdominant species in processes such as soil stabilization and ecosystem resilience are often overlooked (Table 6). To address these gaps, future research should prioritize trait-based approaches to elucidate how specific plant characteristics—such as water-use efficiency, drought tolerance, and root depth—influence ecosystem productivity and services such as soil fertility and carbon storage. Understanding the effects of climate change, including altered temperature and precipitation patterns, on species distribution and community composition is also critical. Additionally, exploring the dynamics of species interactions and their role in ecosystem resilience following disturbances can inform restoration strategies. Applying these insights can enhance sustainable land management practices in arid regions. For instance, selecting climate-resilient species based on functional traits can improve restoration outcomes, while integrating such knowledge into land-use planning can bolster ecosystem services (Table 7). Ultimately, a comprehensive understanding of species and functional group contributions, informed by ongoing research, is essential for the effective management and restoration of desert ecosystems.

**Table 6:** Knowledge gaps and future research directions

Research area	Key questions	Potential applications	Challenges	Future research priorities
Species and functional group dynamics	**China:** How do desert functional groups (e.g., xerophytes, annuals) interact to influence nutrient and water cycling in extreme environments? **Egypt:** how do functional groups respond to salinity and limited water availability in reclaimed and cultivated desert lands?	**China:** improve models for desert ecosystem restoration and conservation planning. **Egypt:** inform land rehabilitation in saline-affected areas and optimize crop choices for arid agriculture.	**China:** limited long-term field data on species turnover and belowground interactions. **Egypt:** salinity, anthropogenic disturbance, and soil degradation confound functional group analysis.	**China:** long-term monitoring of interactions among native functional groups under climate variability. **Egypt:** field trials to examine how functional groups impact soil fertility under irrigation and salinity stress.
Minor species contributions	**China:** what is the ecological role of rare desert shrubs and ephemeral herbs in maintaining microbial and nutrient diversity? **Egypt:** how do subdominant native species contribute to resilience in oasis and marginal desert farming systems?	**China:** promote restoration strategies that maintain rare species and ecosystem function. **Egypt:** design biodiversity-friendly land use in reclaimed deserts.	**China:** rare species are often overlooked in large-scale vegetation assessments. **Egypt:** agricultural focus often leads to neglect of native subdominant species.	**China:** assess contributions of rare species to soil microbial communities and microhabitat stability. **Egypt:** study the role of minor native species in sustainable agriculture and desert greening.
Climate change impacts	**China:** how will increasing temperature and erratic rainfall affect dominant species such as *Haloxylon* and *Reaumuria*? **Egypt:** how will more frequent droughts and heatwaves alter plant community composition in the Western Desert and Nile fringes?	**China:** guide selection of climate-resilient species for arid region afforestation. **Egypt:** support adaptive management for agriculture and ecosystem services under heat/drought stress.	**China:** harsh field conditions make experimental manipulation difficult. **Egypt:** limited climate models specific to desert microclimates.	**China:** use remote sensing and long-term plots to assess functional group shifts. **Egypt:** integrate plant-climate-soil models to predict vegetation change and inform policy.
Species interactions	**China:** how do nurse plants and facilitative interactions drive desert recovery? **Egypt:** what role do native perennials play in rehabilitating degraded soils and promoting annual establishment?	**China:** apply facilitation principles to enhance revegetation success. **Egypt:** design intercropping and agroforestry systems using native perennials.	**China:** disentangling positive vs. competitive interactions is complex in natural systems. **Egypt:** disturbed landscapes reduce potential for native facilitative species.	**China:** study facilitation networks in restored vs. degraded sites. **Egypt:** investigate perennial-annual interactions in desert reclamation contexts.
Trait-based research	**China:** which traits (e.g., rooting depth, seed dormancy, salt tolerance) are key for adaptation to extreme continental deserts? **Egypt:** how do traits such as stomatal regulation and salinity tolerance relate to yield and survival in arid agriculture?	**China:** select native species for functional trait-based restoration. **Egypt:** optimize trait-based breeding of crops and native species for water-limited agriculture.	**China:** trait-environment relationships vary across microhabitats. **Egypt:** trait databases for native plants are underdeveloped.	**China:** link physiological traits with performance under real field conditions. **Egypt:** develop trait libraries and predictive models for sustainable crop and native plant selection.
Sustainable land management	**China:** how can desert-adapted functional groups improve soil structure and fertility over time? **Egypt:** how can combining native species with water-efficient crops reduce land degradation and enhance productivity?	**China:** inform ecological restoration and sand stabilization efforts in northwest China. **Egypt:** enhance sustainability of reclaimed desert lands through ecosystem-based management.	**China:** need for multi-scale approaches integrating soil, vegetation, and land use data. **Egypt:** balancing water scarcity, crop demand, and ecological sustainability.	**China:** develop integrated restoration models using native functional groups. **Egypt:** pilot studies on land use systems combining conservation and productivity goals.

**Table 7:** Summary of key findings and implications

Aspect	Key findings	Implications for China	Implications for Egypt	Global implications
Species contributions	dominant species (e.g., Acacia, Haloxylon) stabilize soil and enhance productivity	focus on cold-tolerant species for restoration	focus on heat-tolerant species for restoration	highlights the importance of species-specific adaptations in arid ecosystems
Functional groups	C4 and CAM plants dominate in arid regions, contributing to water efficiency	promote C4 and CAM plants in restoration projects	promote CAM plants in extremely dry regions	demonstrates the role of functional groups in maintaining productivity under stress
Climate change impacts	rising temperatures and droughts reduce productivity, alter species composition	develop strategies to mitigate seasonal droughts	develop strategies to mitigate prolonged droughts	emphasizes the need for climate-resilient restoration strategies globally
Adaptive mechanisms	deep roots, water storage, and heat tolerance are key adaptations	use deep-rooted species for soil stabilization	use heat-tolerant species for ecosystem resilience	shows how plant adaptations can mitigate climate stress
Management strategies	sustainable agriculture, reforestation, and grazing management are essential	implement rotational grazing and reforestation	implement water-efficient irrigation and grazing management	provides a framework for sustainable land management in arid regions worldwide
Future challenges	balancing economic development with environmental sustainability	address overgrazing and deforestation in cold regions	address water resource exploitation in hot regions	highlights the global challenge of balancing development and conservation
Opportunities	climate-smart agriculture, community-driven conservation, and biotechnology	develop cold-tolerant crops and restoration techniques	develop heat-tolerant crops and water conservation techniques	offers opportunities for global collaboration in arid ecosystem restoration

## Acknowledgments

This research was made possible through the support of the 10.13039/501100012166National Key Research and Development Program of China: Intergovernmental 10.13039/501100018611International Science and Technology Innovation Cooperation Special Project “The Response Mechanisms of Desert Ecosystem Productivity to Extreme Drought in China and Egypt” (project no. 2024YFE0103300).

## Author contributions

W.I.: conceptualization, methodology, investigation, and writing – original draft; B.Z.: conceptualization, validation, formal analysis, and writing – review and editing; B.Y.: software, data curation, and visualization; Q.Y.: resources, investigation, and data curation; H.R.: methodology, validation, and supervision; Y.Z.: formal analysis, writing – review and editing, and project administration; A.A.M.: writing – review and editing, validation, and resources; F.Z.: supervision, funding acquisition, project administration, and writing – review and editing.

## Declaration of interests

The authors have no known competing interests.
